# High-level visual representations in the human brain are aligned with large language models

**DOI:** 10.1038/s42256-025-01072-0

**Published:** 2025-08-07

**Authors:** Adrien Doerig, Tim C. Kietzmann, Emily Allen, Yihan Wu, Thomas Naselaris, Kendrick Kay, Ian Charest

**Affiliations:** 1 Department of Psychology and Education, Freie Universität Berlin, Berlin, Germany; 2 Institute of Cognitive Science, University of Osnabrück, Osnabrück, Germany; 3 Bernstein Center for Computational Neuroscience, Berlin, Germany; 4 Center for Magnetic Resonance Research, Department of Radiology, University of Minnesota, Minneapolis, MN USA; 5 Department of Psychology, University of Minnesota, Minneapolis, MN USA; 6 Graduate Program in Cognitive Science, University of Minnesota, Minneapolis, MN USA; 7 Department of Neuroscience, University of Minnesota, Minneapolis, MN USA; 8 cerebrUM, Département de Psychologie, Université de Montréal, Montreal, Quebec Canada

**Keywords:** Cognitive neuroscience, Neural encoding, Neural encoding

## Abstract

The human brain extracts complex information from visual inputs, including objects, their spatial and semantic interrelations, and their interactions with the environment. However, a quantitative approach for studying this information remains elusive. Here we test whether the contextual information encoded in large language models (LLMs) is beneficial for modelling the complex visual information extracted by the brain from natural scenes. We show that LLM embeddings of scene captions successfully characterize brain activity evoked by viewing the natural scenes. This mapping captures selectivities of different brain areas and is sufficiently robust that accurate scene captions can be reconstructed from brain activity. Using carefully controlled model comparisons, we then proceed to show that the accuracy with which LLM representations match brain representations derives from the ability of LLMs to integrate complex information contained in scene captions beyond that conveyed by individual words. Finally, we train deep neural network models to transform image inputs into LLM representations. Remarkably, these networks learn representations that are better aligned with brain representations than a large number of state-of-the-art alternative models, despite being trained on orders-of-magnitude less data. Overall, our results suggest that LLM embeddings of scene captions provide a representational format that accounts for complex information extracted by the brain from visual inputs.

## Abstract (web summary)

Doerig, Kietzmann and colleagues show that the brain’s response to visual scenes can be modelled using language-based AI representations. By linking brain activity to caption-based embeddings from large language models, the study reveals a way to quantify complex visual understanding.

## Main

The visual system provides the brain with a wealth of information about the physical environment. Much progress in understanding the functional organization^1–5^ and computational principles^6–9^ of the visual system has been driven by a heavy focus on the objects that are present in visual scenes. In particular, exciting advances in the ability to quantitatively predict neural activity in the extrastriate visual cortex have been achieved by training artificial neural networks (ANNs) to perform object recognition from raw visual inputs^10–14^.

Despite this progress, it is clear that visual scenes convey more information than the identity of the objects present^15^. Presumably, an effective interpretation of a visual scene must include the context in which objects reside as well as their spatial and semantic interrelations. Studies of the neural basis of object context and relations have provided insight into the role of object co-occurrence statistics^16,17^, spatial and semantic interrelations among objects^18–21^, the context in which objects appear^22^ and their typical locations in scenes^23–26^. In addition, a robust literature on scene representations in the brain has emerged^27,28^, providing insights into scene categories^27,29–33^, scene grammar^26^ and action affordances^34^, to name a few topics. However, it remains unclear how to connect and integrate the insights obtained from these studies with the kind of quantitative and computational methods (including image-computable models) associated with the object recognition literature. A quantitative approach for studying the complex information extracted from visual scenes seems elusive: what representational format could be used to summarize and study this information?

Excitingly, recent advances in artificial intelligence (AI) provide clues into the challenge of representing scene information. First, large language models (LLMs) have made enormous strides in natural language processing^35^. LLMs learn to encode rich contextual information and statistical world knowledge through training on massive amounts of text data^36–39^. Second, AI researchers have demonstrated improvements in the ability of vision models to segment, recognize and generate images by aligning visual representations with the information conveyed by textual image captions^40–43^. Importantly, these image captions are transformed into a powerful operable format through embedding in the latent space of LLMs^44,45^. These insights lead to an intriguing possibility: LLM embeddings of image captions might be an effective way to capture the rich information conveyed by visual scenes.

In this Article, we explore the hypothesis that the human brain projects visual information from retinal inputs, via a series of hierarchical computations, into a high-level multidimensional space that can be approximated by LLM embeddings of scene captions. To do so, we combine 7 T functional magnetic resonance imaging (fMRI) data collected while participants viewed thousands of natural scenes with multivariate encoding and decoding analyses, as well as ANN modelling. We demonstrate that the visual system may indeed converge, across various higher-level visual regions, towards representations that are aligned with LLM embeddings.

## Results

**Fig. 1:**
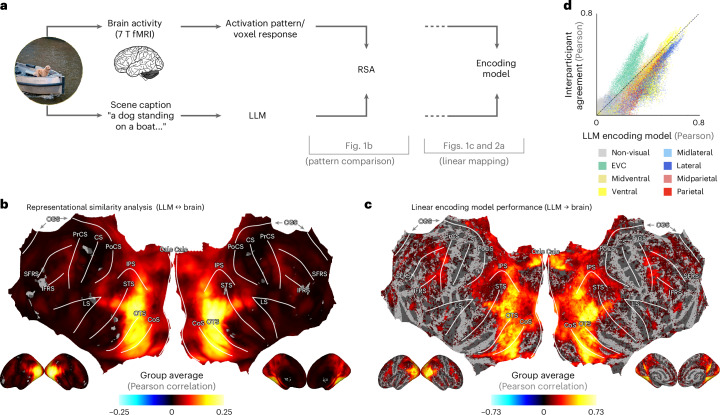
A mapping from LLM embeddings captures visual responses to natural scenes. **a**, LLM to brain mapping methods. Each image in the NSD dataset is associated with captions written by different human observers to describe the scene. These captions are passed through an LLM model to generate embeddings. We use two approaches to quantify the match between these embeddings and fMRI data (RSA and encoding models). Note that, for copyright reasons, we cannot show the real COCO image we used; hence, it has been replaced by a similar copyright-free image. **b**, RSA reveals an extended network of brain regions where LLM representations correlate with brain activities*.* Searchlight map for the group-average Pearson correlation (not noise-ceiling corrected) between LLM embeddings (MPNet) and brain representations (significance threshold set by a two-tailed *t*-test across participants (*N* = 8) with Benjamini–Hochberg false discovery rate (FDR) correction; *P* = 0.05). See Supplementary Fig. 3 for individual participants. **c**, A linear encoding model highlights a similar network of brain regions. We performed voxel-wise linear regression to predict voxel activities from LLM embeddings. Shown is the group-average Pearson correlation map (not noise-ceiling corrected) between the predicted and actual beta responses on the test set (significance threshold set by a two-tailed *t*-test across participants (*N* = 8) with Benjamini–Hochberg false discovery rate correction; *P* = 0.05). See Supplementary Fig. 4 for individual participants. **d**, Encoding model performance versus interparticipant agreement*.* Each dot in the scatter plot shows the encoding model performance for a given voxel versus the interparticipant agreement, computed as the mean Pearson correlation between each participant’s (*N* = 8) voxel activities and the average of the voxel activities of the remaining seven participants on the test images. Our encoding model approaches the interparticipant agreement in all ROIs, indicating good performance. Values below the diagonal can be explained by the fact that the model captures participant-specific variance not captured by the mean of other participants. Calc, calcarine sulcus; CGS, cingulate sulcus; CoS, collateral sulcus; CS, central sulcus; IFRS, inferior frontal sulcus; IPS, intraparietal sulcus; LS, lateral sulcus; OTS, occipitotemporal sulcus; PoCS, post-central sulcus; PrCS, precentral sulcus; SFRS, superior frontal sulcus; STS, superior temporal sulcus.

To explore representational transformations across the visual system, we take advantage of the Natural Scenes Dataset (NSD)^46^, a large-scale 7 T fMRI dataset featuring brain responses to thousands of complex natural scenes taken from the Microsoft Common Objects in Context (COCO) image database^47,48^. The COCO database includes human-supplied captions describing each image, as well as labels for object categories present in each image (see Supplementary Fig. 1 for descriptive statistics of the COCO captions). To test whether LLM embeddings provide a useful representational format for modelling visually evoked brain responses, we use LLM sentence encoders based on transformer architectures^49^ and project the scene captions into the embedding space of these LLMs (Fig. 1a). As a representative LLM, we use MPNet^39^, a transformer that is fine-tuned for sentence-length embeddings. MPNet was chosen as it reaches state-of-the-art performance on a variety of benchmarks, including semantic textual similarity (STS), which measures the match with human judgements of semantic similarity between sentences^50^. Importantly, our LLM embeddings are derived entirely from text, without regard for visual features of the corresponding scenes. This differs from other embeddings that are jointly trained on visual input and language (for example, contrastive language–image pretraining (CLIP)^43^). A two-dimensional *t*-distributed stochastic neighbour embedding (t-SNE) projection of MPNet-embedded NSD captions confirms that the model successfully captures fine-grained scene information, such as what objects are present, what actions are being performed and the type of scene (Supplementary Fig. 2).

### A linear mapping from LLM embeddings captures brain responses to natural scenes

To quantify how well LLM embeddings of scene captions predict brain activities, we used representational similarity analysis (RSA)^4,51–53^. We correlated representational dissimilarity matrices (RDMs) constructed from LLM embeddings of the image captions with RDMs constructed from brain activity patterns obtained while participants viewed the corresponding natural scenes (Fig. 1a). Applying RSA in a searchlight fashion, we find that the LLM embeddings are able to predict visually evoked brain responses across higher level visual areas in the ventral, lateral and parietal streams (Fig. 1b; see Supplementary Fig. 3 for individual participants; see Supplementary Fig. 11 for a reproduction of this result using different LLMs).

We then probed the mapping between LLM representations and brain representations using linear encoding models. We first trained an encoding model to predict individual voxel activities from LLM embeddings using cross-validated fractional ridge regression^54^. In line with the RSA results, we find that the encoding model successfully predicts variance across large parts of the visual system (Fig. 1c,d; see Supplementary Fig. 4 for individual participants). This suggests that the LLM representations of associated captions accurately capture important features of visual processing. We verified that these features generalize across participants by using a cross-participant encoding approach where we train the model on one participant and test it on the other participants (Supplementary Fig. 5).

**Fig. 2:**
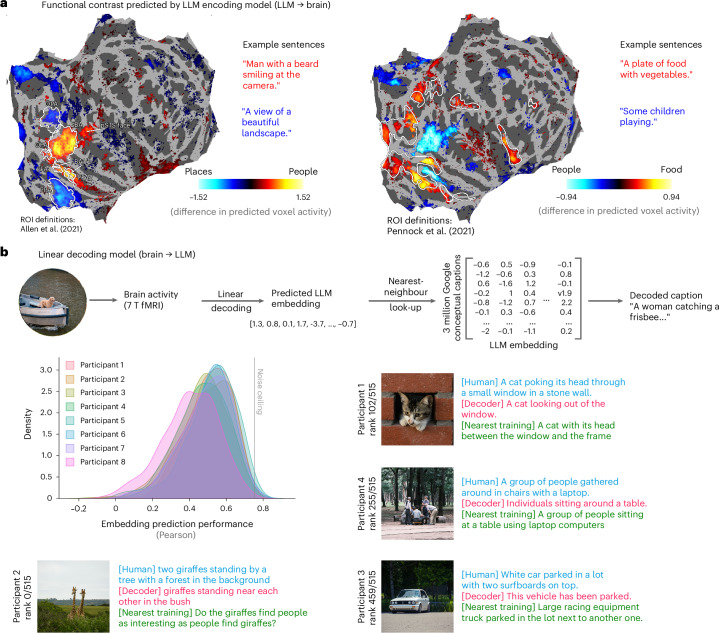
LLM-based linear prediction and decoding of brain activities. **a**, The linear encoding model captures selectivities of different brain regions. We contrasted the brain activity predicted from five novel people- versus place-related sentences (left) and five food- versus people-related sentences (right; significance threshold set by a two-tailed *t*-test across participants (*N* = 8) with *P* = 0.05; without FDR correction). These contrasts highlight brain areas known to be selective for people, places and food (people and place areas are localized as part of NSD (left); food areas described by ref. ^55^ shown as white outlines (right)). **b**, Decoding captions from visually evoked brain responses*.* Top: we fit a linear model to predict LLM embeddings (MPNet) from fMRI voxel activities. We then use a nearest-neighbour look-up to generate a caption for each image. Bottom left: kernel density estimate plot of the prediction score for each participant on a held-out test set (see Supplementary Fig. 5 for a t-SNE projection of the training and testing sets), quantified using Pearson correlation between predicted and target embedding. The noise ceiling is computed as the consistency between the five human-generated captions for each image. Bottom right: target (blue), decoded (pink) and nearest training (green) caption examples from different participants on the held-out test set, spanning the range of prediction scores. The decoder is not simply looking up the closest training item, but instead provides another adequate caption. The rank refers to the prediction score of the shown sample (that is, rank 0 is the best prediction for this participant, while rank 514 is the worst). Note that, for copyright reasons, we cannot show the real COCO images we used; hence, they have been replaced by similar copyright-free images. EBA, extrastriate body area; FBA1/2, posterior/anterior section of fusiform body area; FFA1, posterior section of fusiform face area; FFA2, anterior section of fusiform face area; PPA, parahippocampal place area; pSTS face, posterior superior temporal sulcus face area; OFA, occipital face area; OPA, occipital place area. References: Allen et al.^46^, Pennock et al.^55^.

To elaborate on this point, we tested if the model can reproduce well-established tuning properties observed in cognitive neuroscience. We contrasted the predictions derived from different novel sentences highlighting people versus scenes (for example ‘Man with a beard smiling at the camera’ versus ‘A view of a beautiful landscape’). Such a contrast revealed classical tuning properties associated with people- and place-selective areas (including the fusiform face area (FFA), occipital face area (OFA) and extrastriate body area (EBA) versus parahippocampal place area (PPA) and occipital place area (OPA)) as well as food-selective areas^55^ (Fig. 2a; also see Supplementary Fig. 6). The success of the encoding model indicates that LLM representations, despite being derived purely from text, can make accurate predictions of region-specific tuning properties of the visual cortex.

The success of the LLM representations in characterizing brain activity suggests that it may be possible to accurately infer a textual description of what participants saw from visually evoked brain activity alone using simple linear methods. To test for this, we trained a linear decoding model to predict LLM embeddings from fMRI voxel activities (Fig. 2b). Then, to reconstruct scene captions, we used a dictionary look-up approach^56^ on a large corpus of 3.1 million captions (taken from Google Conceptual Captions^57^). As shown in Fig. 2b, we obtain remarkably accurate textual descriptions of the stimuli viewed by the participants. This highlights the appropriateness of LLM embeddings as a representational format for higher-level brain signals evoked by visual stimuli.

### LLMs integrate complex information contained in scene captions that is important to match brain activities

LLMs are capable of encoding and integrating complex contextual information across all words in scene captions. We hypothesized that this ability can, in part, explain the match of LLM embeddings to brain activities. To test this hypothesis, we contrasted models that differ in their ability to encode contextual information in scene captions. We focused our analyses on regions of interest (ROIs) across the visual system, including early visual cortex (EVC) and the ventral, parietal and lateral visual streams (using the NSD ‘streams’ ROI definitions). We use parameter-free RSA to estimate the representational agreement, and report *t*-test statistics after Benjamini–Hochberg false discovery rate correction with a significance threshold of *P* < 0.05.

**Fig. 3:**
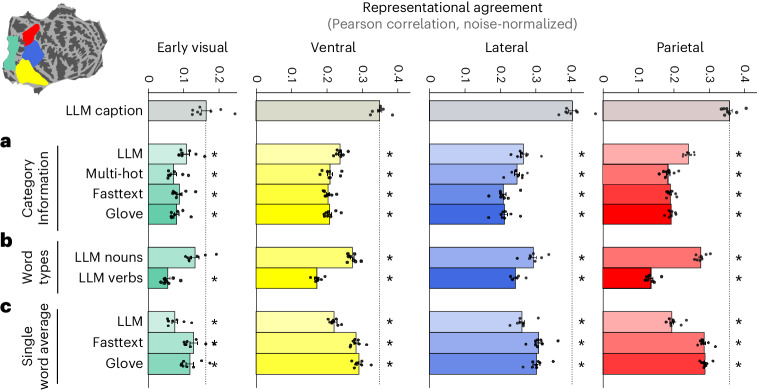
The match of LLMs to visually evoked brain activities derives from their ability to integrate complex information contained in scene captions. We applied RSA in the ‘streams’ ROI definitions of the NSD dataset, shown in the top-left inset. ‘LLM caption’ refers to the LLM embedding (MPNet) of the entire caption, and different groups denote different classes of control models, detailed below. The match between each model and brain activities is quantified as the noise-ceiling-corrected Pearson correlations between each model and a given ROI (averaged across participants (*N* = 8), error bars reflect standard error); all statistics are two-tailed *t*-tests across participants, with Benjamini–Hochberg FDR correction; stars show comparisons where ‘LLM caption’ significantly outperforms the control model (*P* < 0.05); corrected *P* values for all pairwise model comparisons are provided separately in Supplementary Fig. 12. **a**, LLM embeddings of category information improve match to brain data. We compared multiple formats to represent category information, from binary multi-hot vectors (multi-hot), through averaging fasttext (fasttext) or GloVe (glove) word embeddings of category words, to embedding a concatenation of all category words using MPNet (LLM). **b**, LLM embeddings capture brain-relevant information beyond nouns or verbs. The LLM embeddings of the concatenated caption nouns (LLM nouns) or verbs (LLM verbs) both match brain data significantly less well (except LLM nouns in EVC) than the LLM embeddings of the full caption (LLM caption). **c**, LLM embeddings capture brain-relevant contextual information. To test if contextual information conveyed by captions is important to match brain data, we compared embeddings of whole captions with the averaged LLM, fasttext, and GloVe embeddings of individual caption words.

First, we tested if the ability of LLMs to align with high-level visual cortex representations relies on more than just object category information (Fig. 3a). As a base model, we encoded the presence or absence of various object categories using binary multi-hot vectors (as provided by the COCO dataset). We then built increasingly complex models based only on category information: contextually enriched single word embeddings (including fasttext^58,59^, which is based on the context of words, as well as GloVe^60^, which is based on word co-occurence statistics). Such word embeddings provide a richer representation than multi-hot object inventories, because they contain information not only about individual words but also about their typical linguistic context. One step further towards richer, more contextualized representations, we LLM-encoded a concatenated list of all category words. This provides a richer representation of category information, because LLMs can relate and encode interactions between words. LLM embeddings of category words showed significantly better alignment with brain representations than multi-hot vectors (except in the lateral ROI) and word embeddings (except fasttext in EVC). This shows that the LLM representational format allows better predictions of brain activities, even when limited to category information. However, the LLM embeddings of full captions better predicted brain activities in all ROIs by far, indicating that part of the success of LLM mapping to visual brain data is due to its ability to integrate caption information that goes beyond categories. To further test this hypothesis, we conducted the same encoding and decoding analyses as in Fig. 2a,b, but based on LLM embeddings of category words. We found that this leads to worse performance in both analyses, supporting the hypothesis that integrating information beyond categories is important to align LLM and brain representations (Supplementary Fig. 8).

Second, to further understand which aspects of the LLM embeddings drive their agreement with the brain data, we compared LLM embeddings extracted from the full image caption with embeddings obtained from a concatenation of all caption nouns or all caption verbs (Fig. 3b). In agreement with our previous findings, we find that the full caption embeddings significantly outperform the noun- and verb-based embeddings across all ROIs tested, except for noun-based embeddings in EVC. Note that this comparison is a stronger test than the previous analysis of category words, as caption nouns include additional content such as scene locations. Again, this result supports the hypothesis that the brain match of LLM embeddings is driven by the ability to integrate information across the entire captions, beyond nouns or verbs.

We also tested adjectives, adverbs and prepositions, which led to very low alignment with brain representations (Supplementary Fig. 9). This can be expected, given that prepositions, adjectives and adverbs often carry less specific semantic content than nouns and verbs in NSD captions. For example, in the caption ‘a person walking a dog on the grass under a blue sky,’ prepositions like ‘on’ and ‘under’ provide limited predictive information about brain responses. Exploring datasets where these word types play a more important role is an intriguing direction for future research.

Third, we asked whether contextual information between words of a caption is important for the representational match of LLM embeddings with the brain by testing if full caption embeddings provide additional explanatory power beyond that of their constituent words (Fig. 3c). To this end, we compared the LLM caption embeddings with LLM, fasttext and GloVe embeddings averaged across all individual words (that is, these models see all the caption words, but each word is processed separately without the possibility to contextualize one word on the basis of other words in the caption). Again, in all ROIs, the embeddings of whole captions aligned significantly better with brain data than averaged embeddings of the individual caption words. This indicates that the contextual relations among the caption words are an important factor for the LLMs’ alignment with visual representations in the brain.

In further analyses (Supplementary Fig. 10), we generated LLM embeddings from scrambled sentences and found them highly correlated with LLM embeddings from the original sentences (mean Person correlation across eight participants, 0.91; s.d. 0.001). This suggests that the MPNet LLM is relatively insensitive to word order, thus yielding comparable alignment with brain data for both sentence types. While the brain may rely on syntax in language processing, the LLM agreement with visually evoked responses in the brain are not driven by it. Note that scrambled sentences fall outside the LLM’s training distribution, and it may still reconstruct the meaning of the simple NSD captions (for example, it can retrieve the non-scrambled meaning of ‘road a dirt car driving is a on’). This might not happen with more complex sentences where word order is critical. Future research will investigate this further.

Finally, to ensure that our results are not reliant on the exact LLM used for embedding the captions, we tested several other LLMs from the Sentence-Transformers leaderboard (https://www.sbert.net/index.html) and found that they all perform similarly to MPNet used here (Supplementary Fig. 11; none of the statistical comparisons among LLM models was significant). This finding speaks to the generality of our findings and aligns with previous work indicating that scale can matter more than architectural differences in LLMs^61,62^.

### LLM-trained RCNNs outperform other models of visual responses

**Fig. 4:**
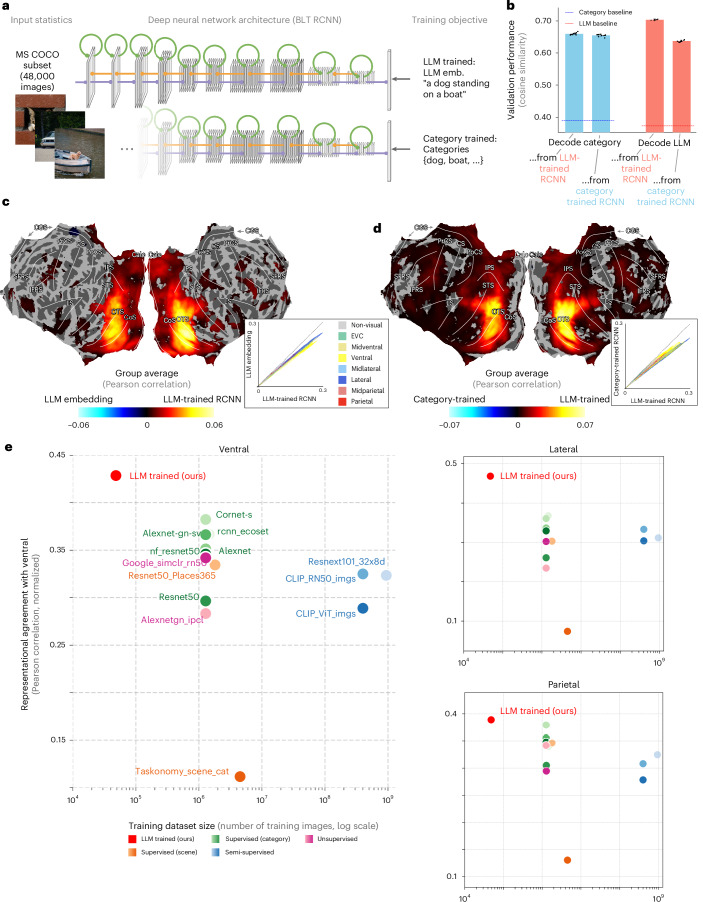
LLM-trained deep recurrent convolutional networks outperform other models in predicting brain activity. **a**, RCNNs. Our RCNNs have ten recurrent convolutional layers with bottom-up (purple), lateral (green) and top-down (orange) connections, followed by a fully connected readout layer. The training objective is to minimize the cosine distance between the network’s output and the target LLM caption embeddings. Category-trained control networks are identical, except that they are trained to predict multi-hot category labels. Note that, for copyright reasons, we cannot show the real COCO images we used; hence, they have been replaced by similar copyright-free images. **b**, Category labels can be decoded from LLM-trained RCNN activities*.* After freezing network weights, we tested how well category labels (respectively LLM embeddings) can be decoded from activities in the pre-readout layer of the LLM-trained (respectively category-trained) network. The plot shows test performance (averaged across *N* = 10 network instances; error bars represent standard deviation), quantified as the cosine similarity between predicted and target vectors. Dashed horizontal bars show floor performance, operationalized as the performance obtained by predicting the mean training target. **c**, LLM-trained RCNNs versus LLM embeddings. Searchlight RSA contrast between llm-trained RCNN activities (last layer and timestep) and the LLM embeddings of scene captions. RCNN RDMs are averaged across ten network instances; correlations are averaged across eight participants; significance threshold set by a two-tailed *t*-test across participants with Benjamini–Hochberg FDR correction; *P* = 0.05. See Supplementary Fig. 15 for individual participants. Insert: brain-model correlation for LLM-trained RCNNs versus LLM embeddings for each searchlight location. **d**, LLM-trained versus category-trained RCNNs. Similar plot as **c**, but showing the contrast between LLM-trained and category-trained RCNNs (last layer and timestep). See Supplementary Fig. 17 for individual participants, Supplementary Fig. 16 for all other RCNN layers and timesteps, and Supplementary Fig. 18 for a reproduction of this effect using the ResNet50 architecture. **e**, ROI-wise comparison of LLM-trained RCNNs with other widely used ANNs. Noise-ceiling-corrected correlations between the pre-readout layer of various models and ROI RDMs. Our RCNN model significantly outperforms all other models (except CORnet-S, which is not significantly worse in the parietal ROI; two-tailed *t*-test across participants with Benjamini–Hochberg FDR correction; *P* = 0.05). Benjamini–Hochberg FDR-corrected *P* values for all pairwise model comparisons are given in Supplementary Fig. 20.

Our results indicate that high-level brain representations are well characterized by LLM-like representations. This leads to the hypothesis the human brain projects visual information from retinal inputs, via a cascade of nonlinear operations across the visual system, into a multidimensional space that can be approximated by LLM embeddings. Under this hypothesis, we predicted that LLM embeddings might serve as a powerful target for training visual ANN models. There has been a history of success using task-optimized ANNs as models of the visual system, but, conventionally, these models are trained to classify objects present in each image^12,13,63,64^ or, in some cases, using unsupervised objectives^65,66^. We therefore trained ANNs to predict LLM embeddings from visual inputs and quantified the match of these task-optimized models to our brain data (Fig. 4a).

We used recurrent convolutional neural networks (RCNNs^67^), based on the vNet architecture^63^ that mirrors the progressive increase of receptive field sizes across the ventral stream. The RCNNs were trained to predict LLM embeddings of the captions associated with the input scenes (LLM-trained RCNNs) on the COCO dataset. To avoid overfitting to the images seen by NSD participants, we excluded from training all images that were used in NSD. We trained ten network instances with different random seeds to account for possible variation due to network initialization^68^. To compare the model response to brain data, we extracted activity patterns in response to the NSD stimuli from the last layer and timestep, computed RDMs and used searchlight RSA to quantify representational alignment with the brain. This searchlight analysis revealed that the LLM-trained RCNN layer activations were able to significantly predict visually evoked brain responses across the entire visual system, similarly to the searchlight performed on the LLM embeddings themselves (Supplementary Fig. 13; see Supplementary Fig. 14 for searchlight maps of all layers and timesteps in the network showing that early layers better match lower visual areas, and higher layers better match higher visual areas).

While results from the previous sections show that high-level brain representations are well captured by visually agnostic LLM embeddings, it is still likely that they retain some visual information that cannot be captured by the LLM embeddings alone (for example, positions of objects that are not explicitly mentioned in the scene captions but are available to inferior temporal cortex^69^). We hypothesized that this information could also be present in the late layers of the LLM-trained RCNN models, which transition from visual inputs to LLM-like representations. In line with this hypothesis, we find that the LLM-trained RCNNs align significantly better with the brain data than the LLM embeddings they were trained to predict (Fig. 4c; see Supplementary Fig. 15 for individual participants). Note that the representations we extracted from our LLM-trained models have 512 features, which is lower-dimensional than the 768 features of the target LLM embeddings. Hence, this result cannot be explained simply by the dimensionality of tested representations.

Despite the strong correlations observed between our LLM-trained RCNNs and the brain data, it is still possible that conventional models trained to classify objects could outperform it. We therefore ran a highly controlled model comparison contrasting our LLM-trained RCNNs with RCNNs trained to predict multi-hot category labels (category-trained RCNNs; again, we trained ten instances with different random seeds). Training these networks end-to-end enabled us to perform a stringent test of our hypothesis: both LLM-trained and category-trained RCNNs are fed the exact same images and have the exact same architecture, the same dimensionality and the same random seeds. They differ only in their training objective (Fig. 4a). To adjudicate between the two models, we contrasted their representational alignment using RSA (focusing on the last layer and timestep activities, as previous work has shown that these layers perform best in predicting higher-level visual regions^63^; searchlight contrast maps between all layers and timesteps of LLM- and category-trained RCNNs can be found in Supplementary Fig. 16). We chose RSA for model adjudication because it provides a robust and unbiased framework for comparing models with varying parameter counts and dimensionalities. By avoiding the need to fit parameters to neural data, RSA ensures that models with more parameters, such as high-dimensional LLM embeddings, do not gain an unfair advantage^70–73^. In line with our hypothesis, the LLM-trained RCNNs significantly outperformed the category-trained controls across a wide network of higher visual areas (Fig. 4d; see Supplementary Fig. 17 for individual participants). The same result was replicated using a ResNet50 (ref. ^74^) architecture, showing that the benefit of LLM training is not restricted to our particular RCNN architecture (Supplementary Fig. 18).

These findings are still consistent with the discovery of object categories as a major factor in ventral stream representations^4,8,75–78^. Indeed, because LLM embeddings capture many forms of linguistically expressible content, LLM representations may encompass content conveyed by object category information. To assess this hypothesis, we froze the weights of our LLM-trained and category-trained RCNNs and quantified how well category labels and LLM embeddings could be linearly read out (Fig. 4b). We found that category labels could successfully be read out from LLM-trained RCNNs (that is, similar performance as for the category-trained RCNNs). However, the reverse was not true: LLM embeddings could not be read out from category-trained RCNNs as well as from LLM-trained RCNNs. These results suggest that the LLM representational format encompasses categorical information while providing a richer training target that improves the match to visually evoked brain activity.

We assessed our LLM-trained RCNNs in the broader landscape of ANN modelling by comparing against 13 models previously reported to be good predictors of visual activity in the brain. These models have diverse architectures, training datasets and objectives and include leading models on neural data prediction benchmarks such as NSD^79^ and brainscore^80^, supervised category-trained models (including a larger version of our RCNN architecture trained on ecoset^63^ and several different models trained on Imagenet^81^), supervised models trained for scene categorization on the Places365 (ref. ^82^) and taskonomy^83^ datasets, weakly supervised models trained on hundreds of millions of images^84^ or image-text pairs (CLIP^43^), and unsupervised models trained using simCLR^85^ and instance-level contrastive learning^65^ (see the Methods for the full list of models). Notably, all of these models are trained on >1 million images (ecoset/ImageNet), or hundreds of millions of images (in the case of resnext101_32x8d_wsl and CLIP), while our LLM-trained RCNN is trained on orders-of-magnitude less data (the 48,000 images left in COCO after removing NSD images).

We applied the same RSA approach as before and report the correlation between each model’s pre-readout RDMs and brain RDMs obtained from higher-level ROIs of the ventral, lateral and parietal visual streams (except for CLIP, for which we used the final embedding instead of the pre-readout layer). We find that our LLM-trained RCNN models, trained to map from pixels to LLM embeddings, significantly outperform every single other model in the ventral and parietal ROIs, and all but one (which is worse, but not significantly) in the lateral ROI (Fig. 4e). To rule out the possibility that this good alignment to brain representations is driven by training on our subset of COCO rather than by the LLM objective, we verified that RCNNs trained to predict category labels on ecoset are not outperformed by our RCNNs trained to predict category labels on our subset of COCO (and both are outperformed by our LLM-trained RCNN; again, we reproduced this result using a ResNet50 architecture; Supplementary Fig. 19). Note that, again, these findings cannot be explained by the fact that LLM-trained models have a higher number of features. First, we use parameter-free RSA, which is not directly biased by feature dimension. Second, the representations extracted from our LLM-trained models have 512 features, which is smaller than most other models (for example, ResNet models have 2,048 features). Together these results suggest that LLM training is a powerful objective to train brain-aligned ANNs. This is in line with the hypothesis that the brain may compute LLM-aligned representations from visual inputs through a cascade of nonlinear computations.

## Discussion

Using a variety of techniques, including RSA, encoding models, linear decoding and ANN modelling, we have provided evidence for the hypothesis that the visual system may converge, across various higher-level regions, towards representations that are aligned with LLM embeddings of captions describing visual inputs. This result is striking, given that LLMs lack any direct visual experience. We suggest that LLM embeddings capture visually evoked brain activity by reflecting the statistical regularities of the world, learned through their extensive language training, in ways that align with sensory processing. In line with this reasoning, we have shown that the success of LLMs in matching brain activities comes from their ability to integrate complex information conveyed by entire captions. The robust and structured mapping between LLM embeddings and visually evoked activities paves the way for new approaches seeking to characterize complex visual information processing in the brain.

Our results build on, and extend, previous research showing the extent of features extracted by visual processing, including object^6–9,86^ and scene^27,29–33^ categories, aspects of linguistics^87,88^, object occurrence statistics^17,33^, the typical location of objects in scenes^23,25^ and many others^22,24,26,27,32,89–98^. Our approach based on LLM embeddings should not be seen as a competitor to these lines of work, but rather as synergistic. While prior work has demonstrated the importance of the above features individually, no unified quantitative framework has been proposed to model them collectively. One exciting avenue of research is to test to which extent LLM embeddings can provide such a unifying quantitative framework. Our work takes initial steps in this direction, as we show that LLM-trained ANNs subsume the category information present in category-trained ANNs. Future work is needed to assess which other known aspects of visual processing are well captured by LLM embeddings.

The success of LLM caption embeddings in predicting high-level visual responses to natural scenes does not imply that these embeddings fully account for the information present in brain responses. Adding access to the actual images seen by the participants can improve prediction performance, as shown by our finding that LLM-trained ANNs taking visual inputs are better aligned with the brain than the LLM caption embeddings. Our interpretation is that the visual system encodes visual input into a representational format that aligns with LLM caption embeddings while retaining some visual information. This interpretation is supported by the good performance of our ANNs, that predict LLM embeddings from visual inputs, compared with a wealth of control models (see refs. ^10,11,99,100^ for discussions of this approach of contrasting ANN models to test computational hypotheses about brain processing).

We find that LLM-trained ANNs outperform a wealth of state-of-the-art neuro-AI models in predicting visually evoked brain activity. This corroborates the hypothesis that the human brain projects visual inputs, through a hierarchy of computations, into a high-level representational format aligned with LLM embeddings of scene captions. One notable aspect of these results is that our LLM-trained ANNs are trained from scratch on orders-of-magnitude fewer images than previous ANN models. Hence, large-scale visual datasets may not be required, if a powerful training objective is used. In this respect, it is important to note that the LLM embeddings themselves are the result of training on large amounts of textual data. Whether these data need to be factored into the training set size estimates is an open question that is beyond the scope of this Article. It may be worth noting, however, that category labels, the gold-standard training objective to which we compare, also rely on substantial amounts of data that went into training the human labelers. An interesting future direction of research will spell out whether the rich learning signal derived from language might indeed provide important benefits over other training objectives, including the supervised, unsupervised, and weakly unsupervised approaches we tested.

Our results do not imply that visual representations have all distinctive attributes of language, such as recursivity and syntax. Rather, what we show is that LLM representations of pure textual input show strong alignment with higher level visual representations, driven by the ability of LLMs to integrate complex information about scenes. These observations open up the possibility that LLM embeddings could be used to predict and decode neural activities in other species that do not have language, such as macaque monkeys^101^. This is in line with recent work in AI, which showed that LLMs can be used to improve the representations of visual models^40–45^, as well as neuroscientific work highlighting similarities between linguistic and visual representations in the brain^102^ and showing that linguistic information improves the ability of crossmodal ANNs to predict brain activities^103,104^.

The task of the NSD participants was to report if they had previously seen each presented image. It cannot be fully ruled out that, to perform this continuous recognition task, participants were internally captioning the scenes, and this may have benefitted the LLM caption embeddings as a good model of visual responses. Alternatively, the brain responses may align well with LLM caption embeddings irrespective of task demands. While data of the scale of NSD are currently not available for other task settings^105^, it will be interesting for future work to investigate LLM-based codes under different tasks. For example, one could use encoding models, as done here, to map from high-level LLM embeddings to visual responses obtained while participants engage in different tasks and investigate the loadings of the linear model on different embedding dimensions^106^.

A representational format that aligns with LLM caption embeddings has potential computational advantages beyond being information-rich, contextual and embedded in world knowledge. Indeed, such rich representations may act as a suitable candidate for communication between different brain systems: if, for example, both visual and auditory processing project to a common (LLM-like) space, information from these modalities can easily be combined and used by other brain processes. Given that LLMs have been shown to be good models for predicting brain activities in language areas too^107,108^, another benefit of this code would be that it may allow easy communication with other organisms^109^.

Our results suggest that the alignment between LLM embeddings and brain responses to visual scenes relies on the rich information encoded in scene captions. This rich information is learned by the LLMs via a series of nonlinear computations converging in a high-dimensional representational space. Although we have begun investigating which aspects of scene captions drive our findings, interpreting LLM embeddings remains a challenging task and is currently an active area of research in explainable AI. Moving forward, additional studies will be essential to clarify which elements of LLM embeddings most strongly correlate with brain representations. At the same time, while developing fully interpretable models of high-level abstract brain processes is an admirable goal, decades of neuroscience research suggest that perfect interpretability may not always be feasible. Indeed, fully interpretable models have historically fallen short of deep neural networks in explaining brain data.

Altogether, our findings indicate that LLM embeddings provide a versatile representational format for capturing the complex information the brain derives from visual inputs. By offering a quantitative, brain-aligned framework, this work paves the way for new research avenues applying modern analysis tools to highly abstract information processed in sensory areas. In the same way that advances in category-based models spurred breakthroughs in visual neuroscience^8,12,13,110^, we anticipate that LLM embeddings—and ANN models capable of extracting such embeddings from visual inputs—will open up fresh directions and yield new insights for both visual computational neuroscience and NeuroAI.

## Methods

### NSD

A detailed description of NSD (http://naturalscenesdataset.org) can be found in ref. ^46^. This dataset contains measurements of fMRI responses from 8 participants who each viewed 9,000–10,000 distinct colour natural scenes over the course of 30–40 scan sessions, comprising a total of 73,000 images, with 3 repetitions per image. Scanning was conducted at 7 T using whole-brain, gradient-echo EPI at 1.8-mm isotropic resolution and 1.6-s repetition time. Images were taken from the COCO image dataset and were presented for 3 s with 1-s gaps in between images. A special set of 1,000 images was shared across participants; the remaining images were unique and mutually exclusive across participants (note that some participants did not complete 3 trials for each image; therefore, only 515 shared images were seen 3 times by all participants). Participants fixated centrally and performed a long-term continuous recognition task on the images. The data were preprocessed by performing one temporal interpolation (to correct for slice time differences) and one spatial interpolation (to correct for head motion) and then using a general linear model to estimate single-trial beta weights. In this Article, we used the 1.8-mm volume preparation of the NSD data (betas_fithrf_GLMdenoise_RR).

### LLM embeddings for NSD stimuli

Captions describing the content of each natural scene were obtained from five human observers as part of the COCO dataset. For each NSD participant and for each image presented to the participant, we gathered the five captions provided for that image and took the mean of the resulting embeddings. This averaging was done to account for interrater differences, which is especially relevant given that the COCO captions were not written by the NSD participants (we ran tests using a single sentence per image with qualitatively similar results (data not shown)). In detail, each of the five captions was passed through an LLM, and we take the average embedding across the captions. For MPNet, we used the all-mpnet-base-v2 version (https://www.sbert.net/docs/pretrained_models.html). Note that this version of MPNet was fine-tuned to have consistent embeddings for different sentences describing the same scene on COCO (on which NSD is based) and other datasets. This ensures that captions written by different people project to a similar point in embedding space, amplifying the ability of the model to extract cross-observer, consistent semantic meaning from captions in the NSD dataset.

In Fig. 3a, we also retrieved the COCO category words for each image (that is, the words associated with the COCO category labels present in the image), concatenated these category words into a string and fed this string into the LLM (called LLM in the figure). In Fig. 3b, we did the same for all nouns and verbs of the captions (using the Natural Language Toolkit (nltk) Python library^111^ to determine which words were nouns and which were verbs, respectively called LLM nouns and LLM verbs in the figure). In Fig. 3c, we also used a single-word-wise LLM embedding (called LLM in the figure). Here, we fed each word from each of the five COCO captions of each image separately into the LLM, and retrieved the average embedding (similarly to how one would use single word embeddings). Finally, in Supplementary Fig. 5, we compared several different LLMs using our standard approach of averaging their embeddings across the five COCO captions.

### Category labels for NSD stimuli

For the multi-hot control in Fig. 3, as well as for training our category-trained ANNs, we used multi-hot binary vectors based on the category labels provided by COCO for each image (that is, vectors of 0 s with 1 s for each category present in the image).

### Word embeddings for NSD stimuli

For word embedding control models (as opposed to the sentence embeddings described above), we used fasttext^58,59^ and GloVe^60^. Using the same COCO image captions as above, we constructed several distinct models, each capturing different aspects of the captions. Word embeddings can be combined additively (a standard example is ‘queen’=‘king’-’man’+’woman’), and so we average the embeddings across words. In Fig. 3a, for category word embeddings, we averaged the word embeddings for each COCO category label. In Fig. 3c, we combined the embeddings for all words in the scene captions by taking the mean embedding across all words of all five COCO captions. Some words were not recognized by fasttext or GloVe because they were either misspelt or did not exist in the corpus. For these cases, we either corrected the misspelling, found a similar word in the fasttext corpus, or removed them. In rare cases, a stimulus may have no category words. In these cases, we used the word embedding for ‘something’ (this is done because every stimulus needs an embedding for RSA, and ‘something’ is a neutral term).

### ANN activations for NSD stimuli

For all ANNs, we collect activities for the layer (and timestep in the case of RCNNs) of interest for all NSD images. We preprocess stimuli to match the input range expected by each model.

### Quantifying model–brain representational agreement using RSA

We used RSA to quantify the match between various models described above and brain representations on the entire NSD dataset. We apply this analysis both ROI-wise (using the ‘streams’ ROI definitions of NSD) and in a searchlight fashion^112,113^.

RDMs were constructed from participants’ native space single-trial beta weights. Analyses were restricted to images that had been seen three times by the participant, and beta weights were *z*-scored across single trials within each scanning session for each participant. We then averaged over each image’s three repetitions to get an average response estimate for each image. In the searchlight analysis, for each voxel *v*, we extracted activity patterns in a sphere centred at *v* with a radius of six voxels (keeping only spheres with more than 50% voxels inside the brain; when a sphere included voxels outside the brain, these voxels were excluded from the analysis). Activity patterns were compared between pairs of stimuli using Pearson correlation distances to create RDMs.

Given the large scale of the NSD dataset, to relate the brain RDMs to the model RDMs, we devised a practical sampling procedure based on independent subsets of images. We first randomly sampled 100 NSD stimuli from the participant’s 10,000 images. We indexed the brain activity patterns for these 100 images and constructed the RDM for this subset. We also indexed the model RDMs to retrieve the pairwise distances for the same 100 stimulus images. This led to 100 × 100 symmetric RDMs, with an upper-triangular vector length of 4,950 pairs (one for each model/RCNN layer and timestep, and one for each ROI/searchlight sphere). These upper-triangular RDMs were then compared between brain and model using Pearson correlation in each ROI/searchlight sphere. There was one such correlation per ROI for each participant–model comparison. The randomly sampled 100 images were then removed from the image sampling pool, and we repeated the sampling procedure until we had exhausted all 10,000 images. This resulted in 100 independent correlation volumes, which were averaged. Note that four participants completed the full NSD experiment, while another two had seen all three repetitions of 6,234 images and two participants had seen the three repetitions of 5,445 images, leading to 100 splits, 62 splits or 54 splits depending on the participant.

For the ROI analyses, each participant’s result was noise corrected. The participant-wise noise ceiling was approximated as the correlation between this participant’s RDM and the mean RDM across all other participants (these RDMs were computed on the shared 515 NSD images seen by all participants). Intuitively, this can be seen as pitting the model against the average of seven human participants: if the model predicts the participant’s data as well as the mean of seven humans, it has reached the noise ceiling. These participant-wise noise-ceiling-corrected correlations were then averaged. Significance was tested using two-tailed *t*-tests across the eight NSD participants, and corrected for multiple comparisons using the Benjamini–Hochberg^114^ procedure for controlling the false discovery rate with *P* = 0.05. For model comparisons, we tested the significance of the difference between model correlations against 0.

For the searchlight analyses, group-level statistics reported in the manuscript are performed using two-tailed *t*-tests across the eight NSD participants and corrected for multiple comparisons using the Benjamini–Hochberg procedure for controlling the false discovery rate with *P* = 0.05. In the case of individual model maps, we tested the model’s correlation against 0. In the case of model comparisons, we tested the significance of the difference between model correlations against 0. Average correlation maps participants, thresholded with our group-level statistics are then projected in freesurfer’s fsaverage surface space and visualized on a flattened cortical flatmap.

### Encoding model

We trained a linear encoding model to predict voxel activities from MPNet embeddings (Fig. 1c). We apply this analysis to the full brain. We used a regularized linear regression framework that was solved for each participant separately. In this framework, the modelled data, **y**, consist of the brain activity measurements (*n* images × *p* voxels) and the predictors, **X**, consist of MPNet embeddings for each image (*n* images × 768 MPNet_dimensions).

We set aside the shared 515 NSD images seen three times by all participants as a test set. We used fractional ridge regression^54^ to estimate the parameters, **ĥ** (*p* voxels × MPNet_dimensions) for 20 different regularization fractions (0.05 to 1 in increments of 0.05), using 5-fold cross-validation. The fraction that best predicted each embedding feature after cross-validation was identified, and used as the final model. To evaluate the model, we computed the Pearson correlation for each voxel between the predicted activities and the true activities on the test set. The group-level statistics reported in the Article are performed using two-tailed *t*-tests across the eight NSD participants, and corrected for multiple comparisons using the Benjamini–Hochberg procedure for controlling the false discovery rate with *P* = 0.05.

### Encoding-model-based brain activity predictions

Our encoding model allows us to predict the brain activities from any sentence. That is, we can predict the activities that would be evoked if the participant saw an image captioned by that sentence. To this end, we simply write a sentence, project it in LLM embedding space and use the resulting embedding as input to our encoding model. To test this approach, we reproduced contrasts from the neuroscientific literature (Fig. 2a). In each contrast, we write five sentences for each group, average the predicted activities and plot the contrast between these activities on brain maps (unlike all other maps in this Article, there is no correction for false discovery rate). We did not have a precise method for selecting these sentences, and simply attempted to make them representative of the contrasts we aimed to reproduce. The sentences we used for each contrast are shown below.

- **People**
  - ’Man with a beard smiling at the camera.’
  - ’Some children playing.’
  - ’Her face was beautiful.’
  - ’Woman and her daughter playing.’
  - ’Close up of a face of young boy.
- **Places**
  - ’A view of a beautiful landscape.’
  - ’Houses along a street.’
  - ’City skyline with blue sky.’
  - ’Woodlands in the morning.’
  - ’A park with bushes and trees in the distance.’
- **Food**
  - ’A plate of food with vegetables.’
  - ’A hamburger with fries.’
  - ’A bowl of fruit.’
  - ’A plate of spaghetti.’
  - ’A bowl of soup.

### Decoding of LLM embeddings from brain data

We decoded captions from visually evoked activity by learning a linear mapping from brain activity to the LLM caption embeddings (this mapping can be seen as the inverse mapping to the encoding model described above), followed by a dictionary look-up scheme^56^ (Fig. 2b).

We apply this analysis on all voxels inside the’streams’ visual ROIs (provided by NSD). We used a regularized linear regression framework that was solved for each participant separately. In this framework, the modelled data, **y**, consist of the captions embeddings (*n* images × 768 MPNet_dimensions) and the predictors, **X**, consists of brain activity measurements (*n* images × *p* voxels).

We set aside a test set to test the performance of the decoding, by holding out the shared 515 NSD images seen three times by all participants. We used fractional ridge regression^54^ to estimate the parameters, ${\hat{\mathbf{h}}}$ (*p* voxels × 768 MPNet_dimensions), that represent the optimal sets of weights to apply to the predictors (**X**_**train**_) to best predict each of the captions embedding features (**y**). Specifically, weights were estimated for 20 different regularization fractions (0.05 to 1 in increments of 0.05), using 5-fold cross-validation. The fraction that best predicted each embedding feature after cross-validation was identified, and the resulting model was evaluated on the test set by using the corresponding weights to predict the captions embeddings.

To quantify the accuracy of our test predictions, we compared the Pearson correlation between the predicted embedding and the target test embedding and plotted a participant-wise kernel density estimate of these correlations. As a noise ceiling, we use the internal consistency of the five human-generated captions in COCO. To this end, we compute the Pearson correlation between the LLM embeddings of each of the five captions and the averaged embedding of the four others and average the resulting five correlations.

To obtain a caption reconstruction, we used a simple dictionary look-up scheme. We took the 3.1 million captions from the Google conceptual captions dataset^57^ and embedded these captions using MPNet, yielding a look-up dictionary **D** with dimensionality 3.1 million captions × 768 MPNet_dimensions. For each embedding predicted from the brain data, we computed the Pearson correlation with each of the captions in the dictionary. The caption that was closest to the predicted embedding was chosen as the reconstructed caption.

### RCNNs

Our RCNN models are derived from vNet, a ten-layer convolutional deep neural network architecture designed to closely mirror the progressive increase in foveal receptive field sizes found along the human ventral stream, as estimated by population receptive fields^63^. In contrast to previous instances of vNet, our network is recurrent, including both lateral and top-down recurrent connections following a convolutional pattern, as implemented by Kietzmann et al.^115^.

We used the COCO dataset for training. As the NSD dataset is based on a subset of COCO, we removed the 73,000 images of the NSD dataset from the training and validation sets, and used them as our testing set (that is, the networks did not see any of the NSD images during training, nor in validation). This resulted in 48,236 COCO images for training, 2,051 for validation and the 73,000 images part of both COCO and NSD for testing. For rectangle images, we took the largest possible square crop, as was done for the NSD experimental stimuli. Images were resized to 128 × 128 pixels.

We trained our recurrent vNet to map from pixels, that is, COCO images, to LLM embeddings (that is, MPNet embeddings of COCO captions extracted as described in ‘LLM embeddings for NSD stimuli’ section). The readout layer therefore was 768-dimensional, to match MPNet embeddings (we did not apply a traditional nonlinearity softmax or sigmoid activation function to the readout, as MPNet embeddings can be both positive and negative). The objective of the network was to minimize the cosine distance between the predicted and the target LLM embedding. To account for possible variation due to the network randomly initialized parameters, we trained ten instances with different random seeds^68^.

As a stringently controlled comparison model, we trained a separate vNet with identical architecture on a category objective (that is, minimizing cosine distance using a multi-hot encoding of the category labels provided in the COCO dataset for each image, this model has a sigmoid activation function, as is usual for multiclass categorization). Again, we trained ten instances with different random seeds.

To show that the advantage of training on LLM embeddings is not restricted to this current RCNN architecture, we reproduced these results using a ResNet50^74^ architecture instead of our RCNNs (one seed each). We used non-pretrained ResNet50, which we trained to predict either LLM embeddings or category labels, as we did for our RCNNs.

All networks were trained using an Adam optimizer with a learning rate of 5 × 10^−2^ and an epsilon of 1 × 10^−1^ for 200 epochs, with a warm-up phase of 10 epochs where the learning rate was linearly increased, followed by a cosine decay. We used a batch size of 96 for RCNNs and 512 for ResNets.

### RCNN fine-tuning

To test if category labels (respectively LLM embeddings) can be decoded from LLM-trained (respectively category-trained) RCNN activities, we performed fine-tuning experiments. We collected activities for the last layer and timestep from each of the ten instances of each network on the entire NSD dataset (collecting activities in this way is equivalent to freezing the weights of the network but does not require recomputing the activations at each epoch). We used the first 71,000 images of NSD as a training set and set aside the last 2,000 as a test set. We trained linear readouts to decode multi-hot category labels (respectively LLM embeddings) from the activities of LLM-trained (respectively category-trained) networks, by minimizing the cosine distance between prediction and target (as described above for training the full RCNN; the readout activation, optimizer and training hyperparameters were also the same as for training the full RCNN). We then average the test performance across the ten network instances with different seeds. As a noise floor, we computed the mean LLM embedding (respectively multi-hot vector) across the 48,238 images used to train the RCNNs and computed the mean cosine distance with the LLM embedding (respectively multi-hot vector) of the 2,051 validation images.

### Other ANNs

We tested several other ANNs. These include:

#### Supervised models (object category)

- We trained an RCNN on object classification on the ecoset dataset^63^. To help the network deal with this larger dataset, we doubled the number of channels. Otherwise this network was identical to the previous RCNNs.
- CORnet-S^116^ trained on imagenet^81^, taken from thingsvision^117^.
- Alexnet^118^ trained on imagenet, taken from brainscore^80^.
- Alexnet-gn trained on imagenet, taken from ref. ^65^.
- resnet50 trained on imagenet, taken from brainscore.
- Nf-resnet50 trained on imagenet (best-performing CNN on predicting NSD data in ref. ^79^, taken from timm^119^).

#### Supervised models (scene category)

- We trained a ResnNet50 trained on scene categorization on the places365 dataset^82^.
- A ResNet50 trained on scene categorization on the taskonomy dataset^83^, taken from https://github.com/StanfordVL/taskonomy.

#### Semi-Supervised models

- Resnext101_32x8d_wsl^84^, trained on 914 million public images (best brainscore model available to download), taken from https://pytorch.org/hub/facebookresearch_WSL-Images_resnext/.
- CLIP_RN50_imgs (that is, the visual stream of CLIP with a ResNet50 backbone)^43^, trained on webimagetext^43^.
- CLIP_ViT (that is, the visual stream of CLIP with a vision transformer backbone), trained on webimagetext, taken from https://github.com/openai/CLIP.

#### Unsupervised models

- Alexnet, trained using instance-prototype contrastive learning on imagenet, taken from ref. ^65^.
- ResNet50, trained using SimCLR^85^ on imagenet, taken from https://github.com/google-research/simclr.

### Predicting brain activity from ANN activations

To compare the representations in our networks to the brain’s representations, we apply a similar RSA approach as described above. First, RDMs for all images in the NSD dataset are computed in the layer (and timestep) of interest in the networks. Second, correlations between RCNNs and brain RDMs are computed, ROI-wise or in a searchight fashion. To quantify how well layer *L* at timestep *T* predicts brain activity, we computed the Pearson correlation between the RDM for layer *L* at timestep *T* and the brain data RDM at each ROI or searchlight location. In the case of our RCNNs, for which we have ten instances with different random seeds, we compute individual RDMs for each seed and then average correlations with brain data across seeds.

### Reporting summary

Further information on research design is available in the Nature Portfolio Reporting Summary linked to this article.

## Supplementary information

Supplementary Information Supplementary Figs. 1–20.

Reporting Summary

## Data Availability

The Natural Scenes Dataset is available at http://naturalscenesdataset.org.

## Code availability

Code for the analyses reported here is available via GitHub at https://github.com/adriendoerig/visuo_llm (ref. ^120^).
