# Supplementary material for: High-level visual representations in the human brain are aligned with large language models
Source: Nat Mach Intell. 2025 Aug 7;7(8):1220–34. doi: 10.1038/s42256-025-01072-0 (PMC12364710; doi:10.1038/s42256-025-01072-0)
Supplement: Supplementary file 1 — Supplementary Figs. 1–20. [file 42256_2025_1072_MOESM1_ESM.pdf]

nature machine intelligence

Supplementary information https://doi.org/10.1038/s42256-025-01072-0

High-level visual representations in the human brain are aligned with large language models

In the format provided by the authors and unedited

High-level visual representations in the human brain are aligned with large language models

Supplementary Information

Table of contents:

Supp. Figure 1: Descriptive statistics of COCO captions.
Supp. Figure 2: Visualising LLM embeddings space for NSD.
Supp. Figure 3: MPNet-brain RSA correlation at the group- and subject-level.
Supp. Figure 4: MPNet encoding model performance at the group- and subject-level.
Supp. Figure 5: Cross-subject MPNet encoding models.
Supp. Figure 6: Predicting neuroscientific contrasts from single sentences.
Supp. Figure 7: T-SNE projection of the training and testing sets used for encoding and decoding models (subject 1)
Supp. Figure 8: Integrating information beyond categories is important for good encoding and decoding performance.
Supp. Figure 9: MPNet representational alignment with visual brain areas by word type.
Supp. Figure 10: Effect of word order scrambling on MPNet representational alignment with visual brain areas.
Supp. Figure 11: Different LLMs all match visually-evoked brain activities similarly well.
Supp. Figure 12: All Benjamin & Hochstein false discovery rate corrected p-values for Figure 3.
Supp. Figure 13: LLM-trained RCNN searchlight at the group- and subject-level.
Supp. Figure 14: LLM-trained RCNN searchlight for all layers and timesteps.
Supp. Figure 15: LLM-trained RCNN vs. LLM searchlight at the group- and subject-level.
Supp. Figure 16: LLM-trained vs. category-trained RCNN searchlight contrasts for all layers and timesteps.
Supp. Figure 17: LLM-trained RCNN vs. category-trained RCNN searchlight at the group- and subject-level.
Supp. Figure 18: Reproducing the benefit in brain alignment of LLM training using ResNet50.
Supp. Figure 19: The advantage in brain alignment of our LLM-trained models is due to their training objective and not to the dataset.
Supp. Figure 20: All Benjamin & Hochstein false discovery rate corrected p-values for Figure 4E.

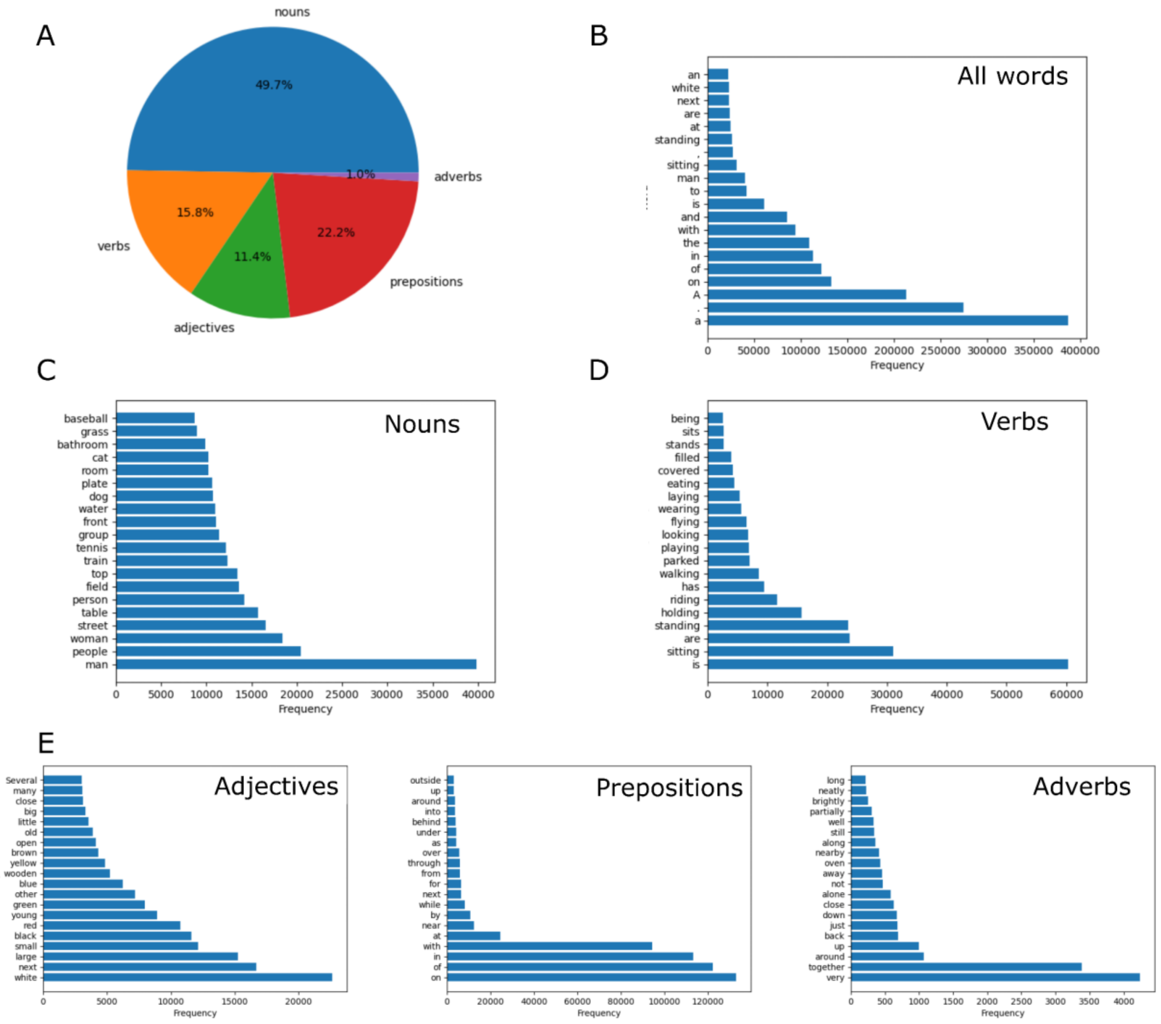

Supp. Figure 1: Descriptive statistics of COCO captions. A: Frequencies by word type. **B.** Most frequent words. **C-E.** Most frequent words by word type.

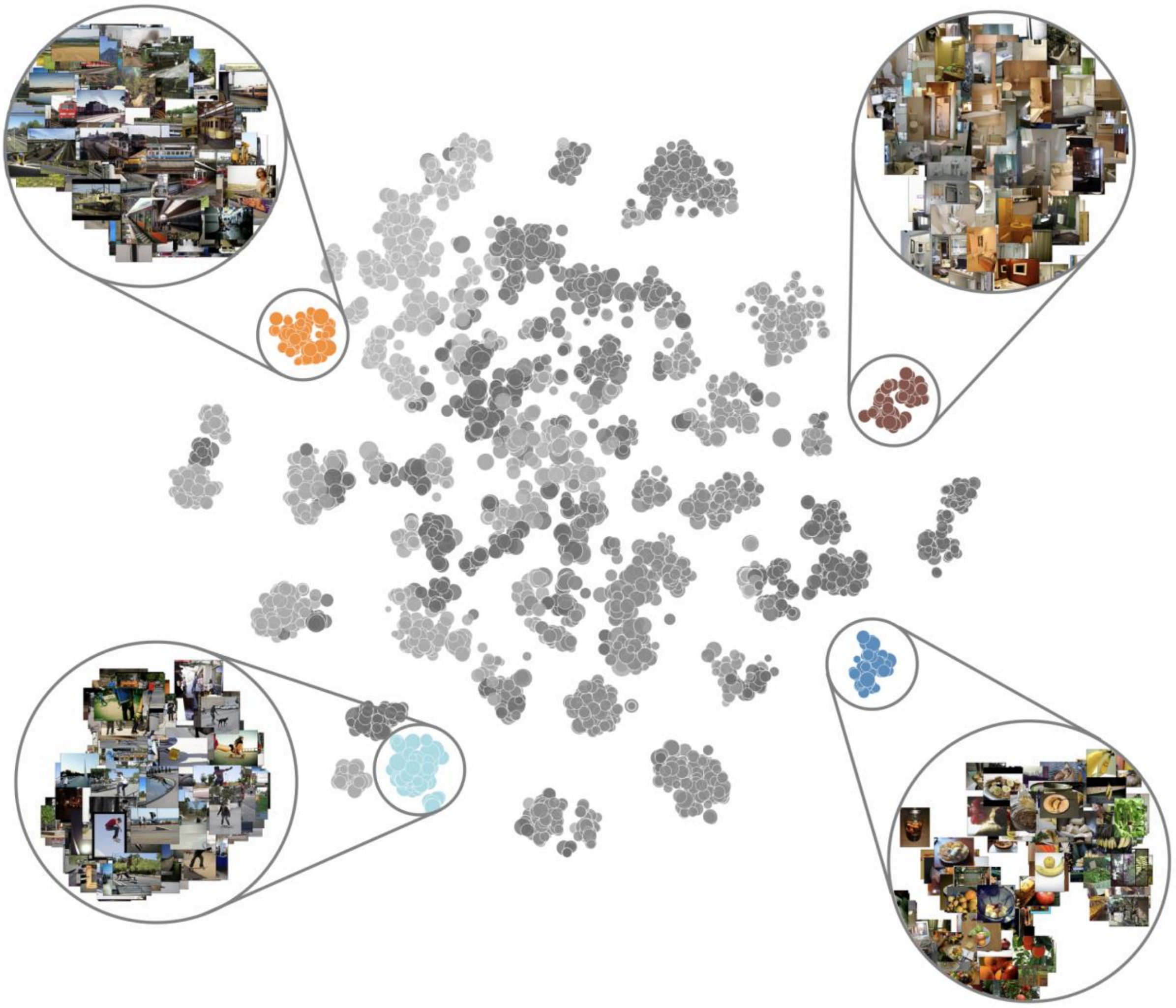

Supp. Figure 2: Visualising LLM embeddings space for NSD. 2D T-SNE projection of the MPNet embeddings for the NSD dataset images seen by participant 1. Note that although each element in the dataset is represented here by the associated image for interpretability, T-SNE is computed on MPNet embeddings of the image captions. These embeddings capture relevant semantic information, as shown by the semantically related clusters.

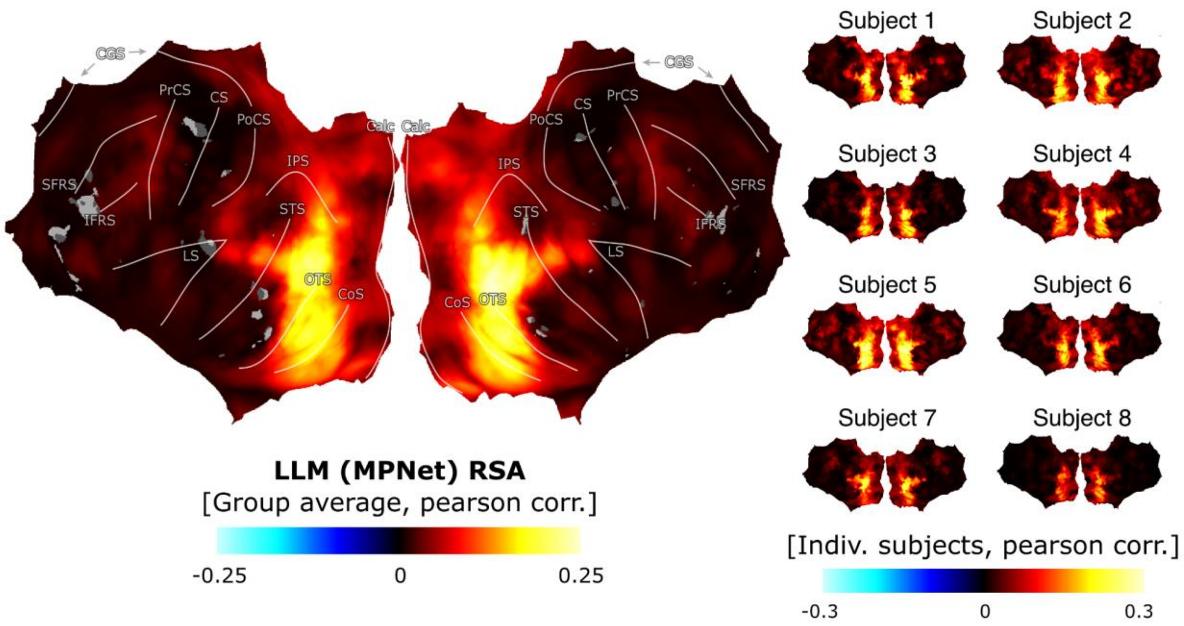

Supp. Figure 3: MPNet-brain RSA correlation at the group- and subject-level. This is an extension of Fig. 1B. *Left*: Searchlight map for the Pearson correlation (not noise-ceiling corrected) between LLM embedding space (as given by MPNet embeddings) and brain representational space. Left: Group average (significance threshold set by a 2-tailed *t*-test across subjects (N=8) with Benjamini & Hochberg false discovery rate correction; $p = 0.05$). *Right*: individual subjects, not thresholded for significance.

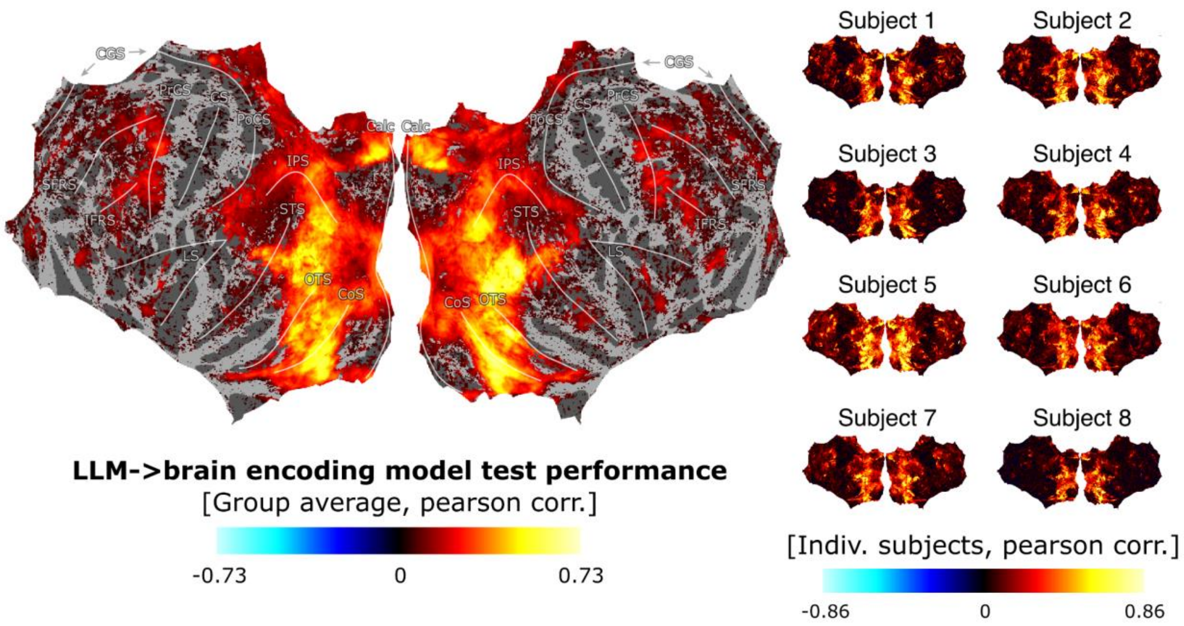

Supp. Figure 4: MPNet encoding model performance at the group- and subject-level. This is an extension of Fig. 1C. *Left*: Pearson correlation map (not noise-ceiling corrected) between the predicted beta responses on a held out test set, and the actual observed beta responses on this test set, averaged across subjects (N=8; same as Fig. 1C; significance threshold set by a 2-tailed t-test across subjects with Benjamini & Hochberg false discovery rate correction; p=0.05). *Right*: individual subjects, not thresholded for significance.

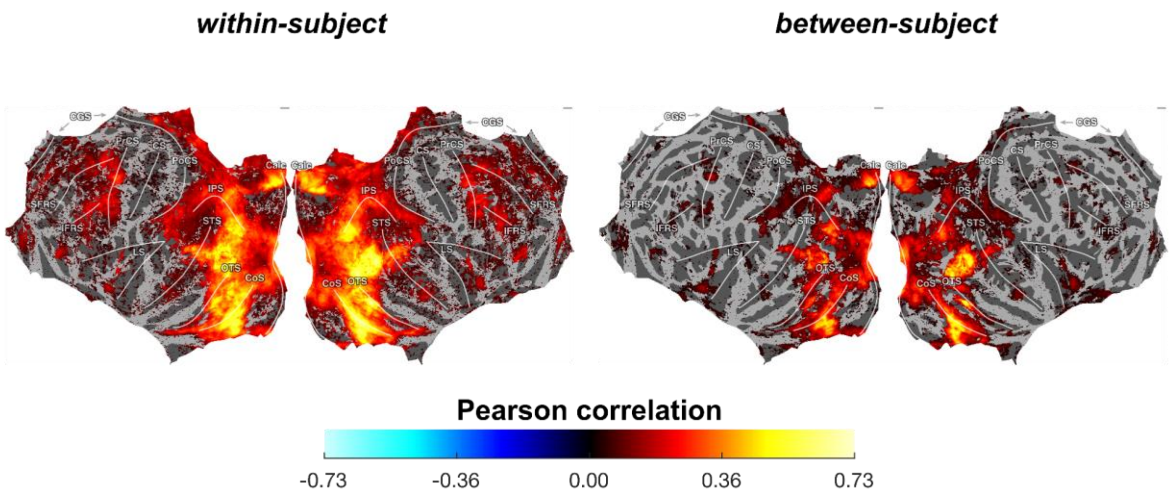

Supp. Figure 5: Cross-subject MPNet encoding models. *Left:* Within-subject encoding model performance (not noise-ceiling corrected; same as Fig. 1C). *Right:* Between-subject encoding model performance. This map is similar to the left panel, but instead of training and testing the encoding model on the same subject, each encoding model is trained on one subject, and tested on all other subjects. We computed the Pearson correlation (not noise-ceiling corrected) between the predicted beta responses on a held out test set, and the actual observed beta responses of other subjects on this test set for every vertex. This yields 7 maps per subject (i.e., one map for predicting each other subject from the encoding model trained on this subject, for a total of 8 subjects * 7 maps_per_subject = 56 maps. We plot the average of these 56 maps, and significance threshold is set by a 2-tailed t-test across these 56 maps with Benjamini & Hochberg false discovery rate correction; p=0.05).

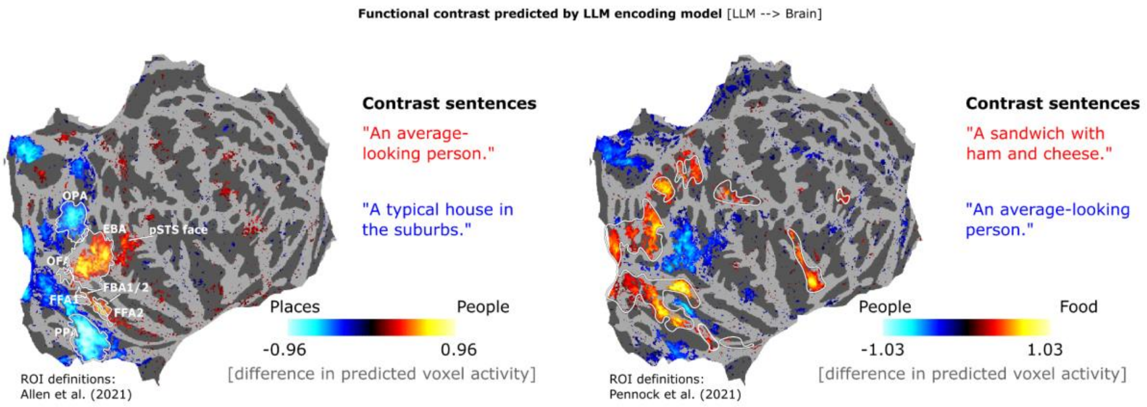

Supp. Figure 6: Predicting neuroscientific contrasts from single sentences. We reproduced our results from Fig. 2A using contrasts of brain activities predicted from individual sentences. We used different sentences than the ones from Fig. 2A. For each contrast, we write two sentences, obtain the predicted activities, and plot the contrast between these predicted activities on brain maps. The sentences we used for each contrast were ’An average-looking person.’ vs. ’A typical house in the suburbs.’ for people vs. places (left), and ’A cheese and ham sandwich.’ vs. ’An average-looking person.’ for food vs. people (right; significance threshold set by a 2-tailed t-test across subjects (N=8) with p=0.05; no correction for false discovery rate was performed). The predicted contrasts reproduce previously described contrasts in the neuroscientific literature (previously described people, place, and food areas are shown as overlays, as in Fig. 2A).

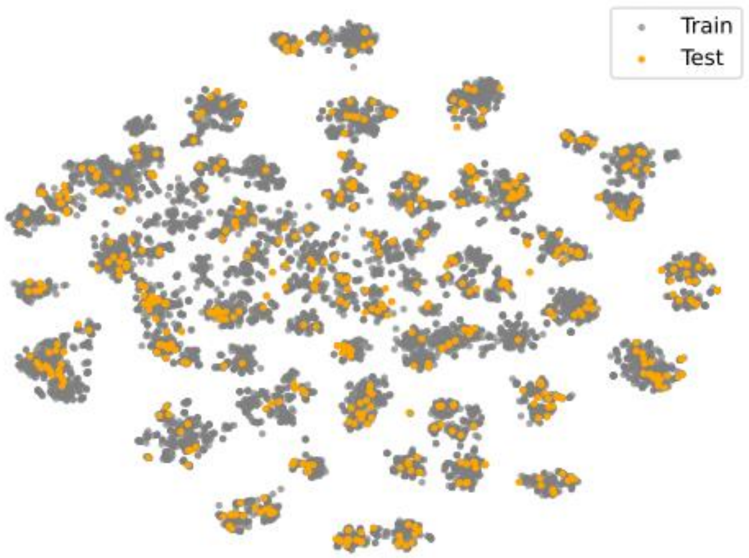

Supp. Figure 7: T-SNE projection of the training and testing sets used for encoding and decoding models (subject 1). The test examples are spread over the training distribution, as is standard in machine learning looking for in-distribution generalisation rather than 0-shot transfer to out of distribution (i.e. the same would hold true for ILSVRC training/test sets for example).

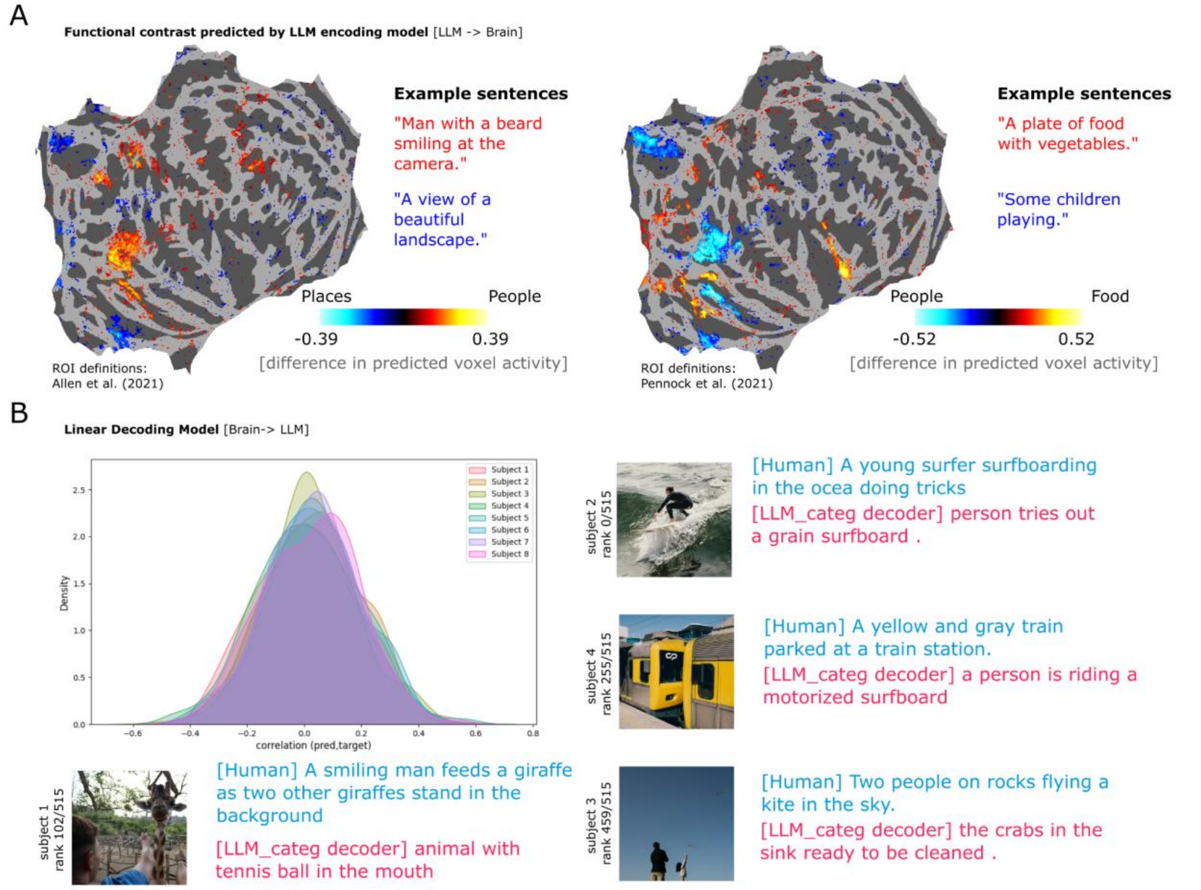

Supp. Figure 8: Integrating information beyond categories is important for good encoding and decoding performance. A. Maps are shown as in Fig. 2A. **B.** Results are shown as in Fig. 2B. Note that, for copyright reasons, we cannot show the real COCO images we used, hence they have been replaced by similar copyright-free images

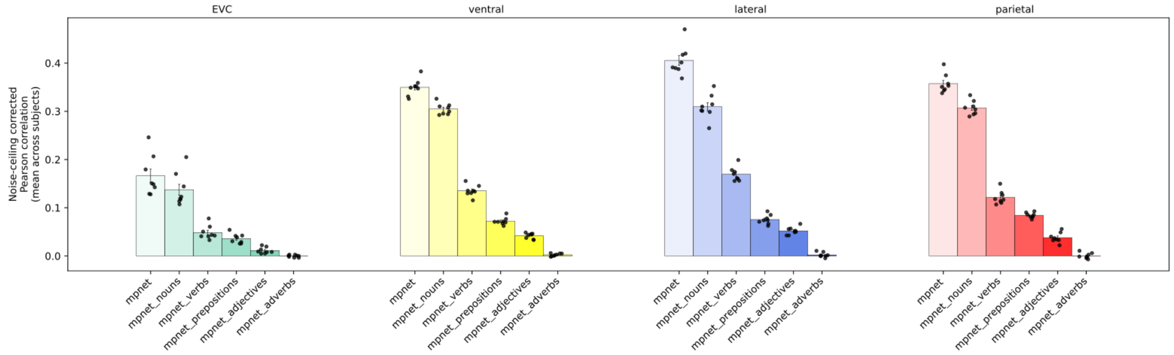

Supp. Figure 9: MPNet representational alignment with visual brain areas by word type. We compared the brain alignment of MPNet embeddings of full scene captions (mpnet), with the brain alignment of MPNet embeddings of captions restricted to various word types (for example, mpnet_adjectives denotes MPNet embeddings of the concatenated adjectives of each caption). We applied our RSA approach in the 'streams' ROI definitions of the NSD dataset, shown in the insert of Fig. 3. The colours of the bars in the plot are colour-coded to match the insert. The x-axis labels denote model names. The match between each model and brain activities is quantified as the noise ceiling corrected correlations between RDMs for each model and a given ROI (averaged across 8 subjects; error bars reflect standard error across subjects). The low performance of prepositions, adjectives, and adverbs can be expected because, in the NSD captions, these word types often carry less specific semantic content compared to nouns and verbs (cf. also Supp. Fig. 1). For instance, if the caption is 'a person walking a dog on the grass under a blue sky,' using only the prepositions 'on' and 'under' would presumably not capture the relevant information necessary to predict brain responses effectively. Applying our framework to datasets where propositions, adjectives and/or adverbs play a stronger role than they do in NSD is an interesting future direction of research.

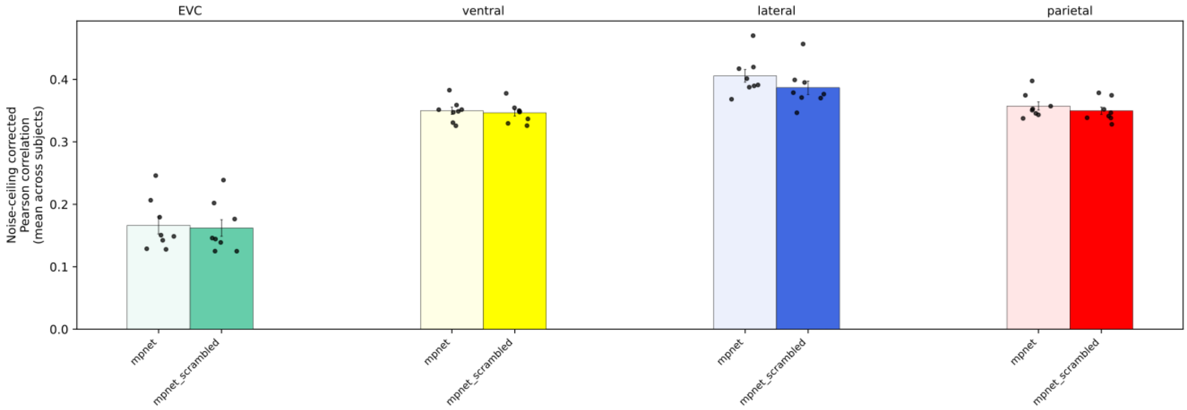

Supp. Figure 10: Effect of word order scrambling on MPNet representational alignment with visual brain areas. We compared the brain alignment of MPNet embeddings of scene captions, with the brain alignment of MPNet embeddings of scrambled scene captions (i.e., we scrambled word order before retrieving the embeddings). We applied our RSA approach in the 'streams' ROI definitions of the NSD dataset, shown in the insert of Fig. 3. The colours of the bars in the plot are colour-coded to match the insert. The x-axis labels denote model names. The match between each model and brain activities is quantified as the noise ceiling corrected correlations between RDMs for each model and a given ROI (averages across 8 subjects; error bars reflect standard error across subjects). None of the statistical comparisons were significant (2-tailed *t*-test across participants, $p>0.05$, Benjamin & Hochstein false discovery rate corrected). Note that the embeddings of scrambled and non-scrambled captions were highly correlate (Pearson r = 0.91). Therefore, these results indicate that the MPNet embeddings are not strongly affected by word order.

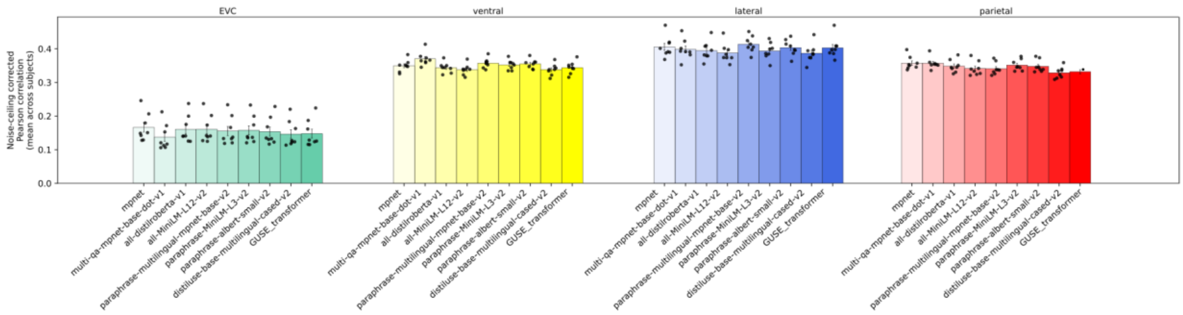

Supp. Figure 11: Different LLMs all match visually-evoked brain activities similarly well. To ensure that our results are not reliant on the specific LLM used for embedding the captions, we compared 9 LLMs from the Sentence-Transformers leaderboard (https://www.sbert.net/index.html). We applied our RSA approach in the 'streams' ROI definitions of the NSD dataset, shown in the insert of Fig. 3. The colours of the bars in the plot are colour-coded to match the insert. The x-axis labels denote model names. The match between each model and brain activities is quantified as the noise ceiling corrected correlations between RDMs for each model and a given ROI (averaged across subjects; error bars reflect standard error across subjects). None of the statistical comparisons among LLM models were found to be significant (2-tailed *t*-test across participants, $p>0.05$, Benjamin & Hochstein FDR corrected). This finding speaks for the generality of our findings, and aligns with previous work indicating that scale can matter more than architectural differences in LLMs[61,62].

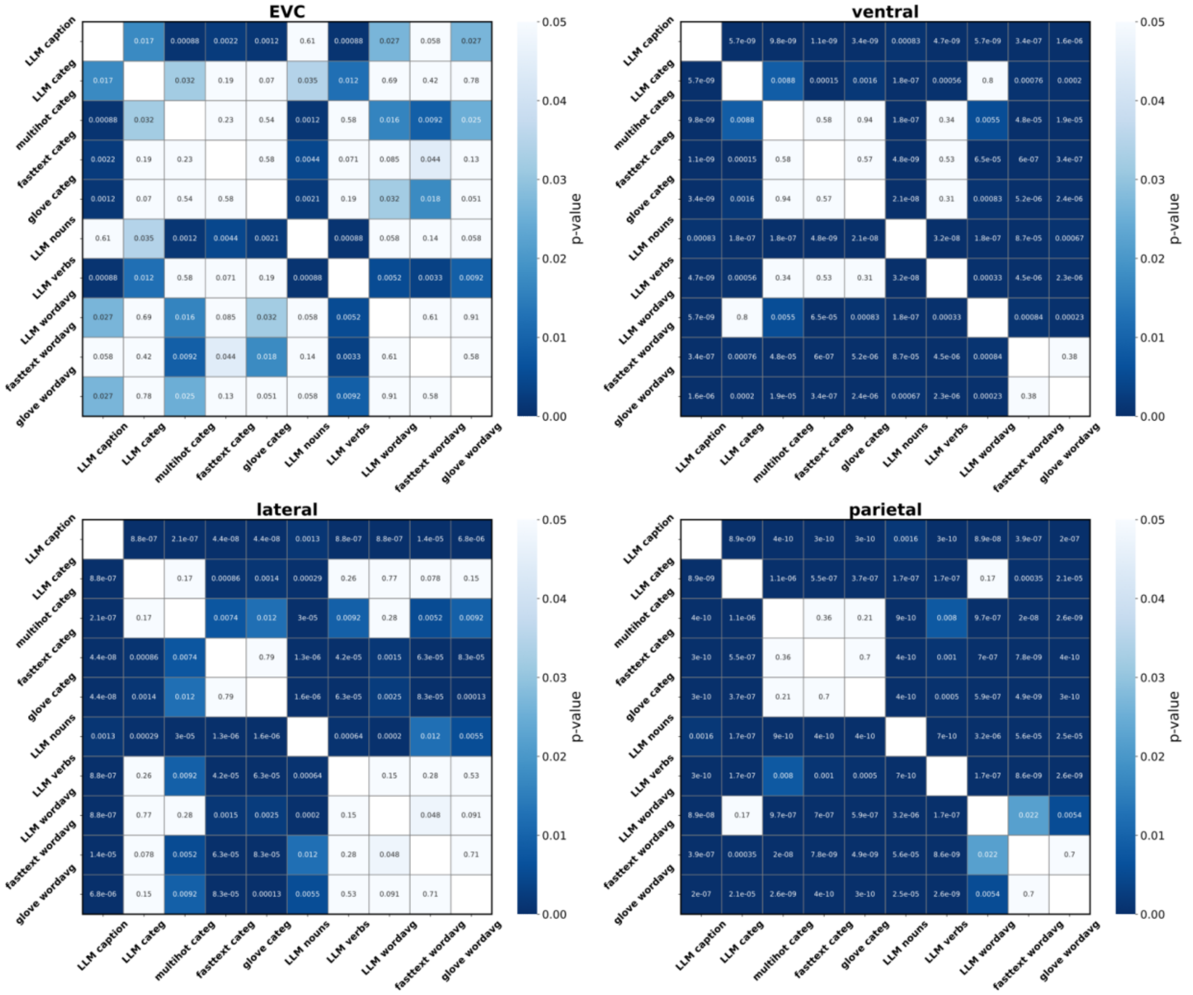

Supp. Figure 12: All Benjamin & Hochstein false discovery rate corrected p-values for Figure 3. 2-tailed *t*-test across the 8 NSD participants. Corrections are conducted across all models, independently for each ROI. Model names are listed in the same order as in Fig. 3.

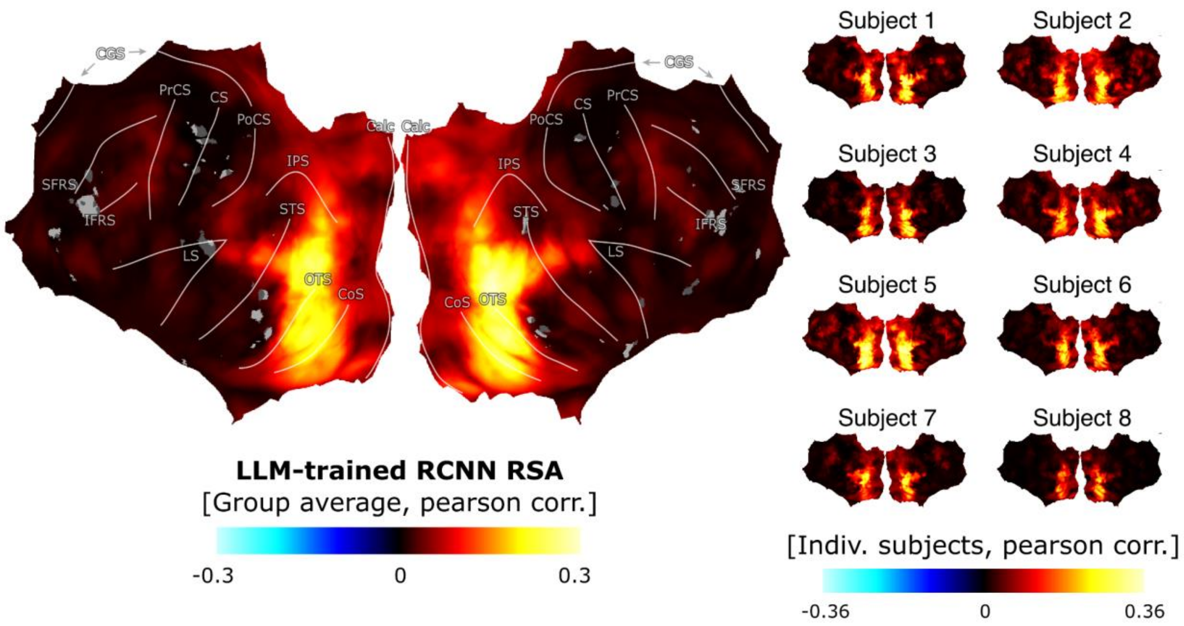

Supp. Figure 13: LLM-trained RCNN searchlight at the group- and subject-level. *Left*: Searchlight map for the correlation between LLM-trained RCNN representational space (last layer and timestep) and brain representational space. RCNN RDMs are averaged across 10 network instances; Pearson correlations (not noise-ceiling corrected) are averaged across 8 participants for the group average; significance threshold set by a 2-tailed *t*-test across subjects (N=8) with Benjamini & Hochberg false discovery rate correction; p = 0.05). *Right*: individual subjects, not thresholded for significance

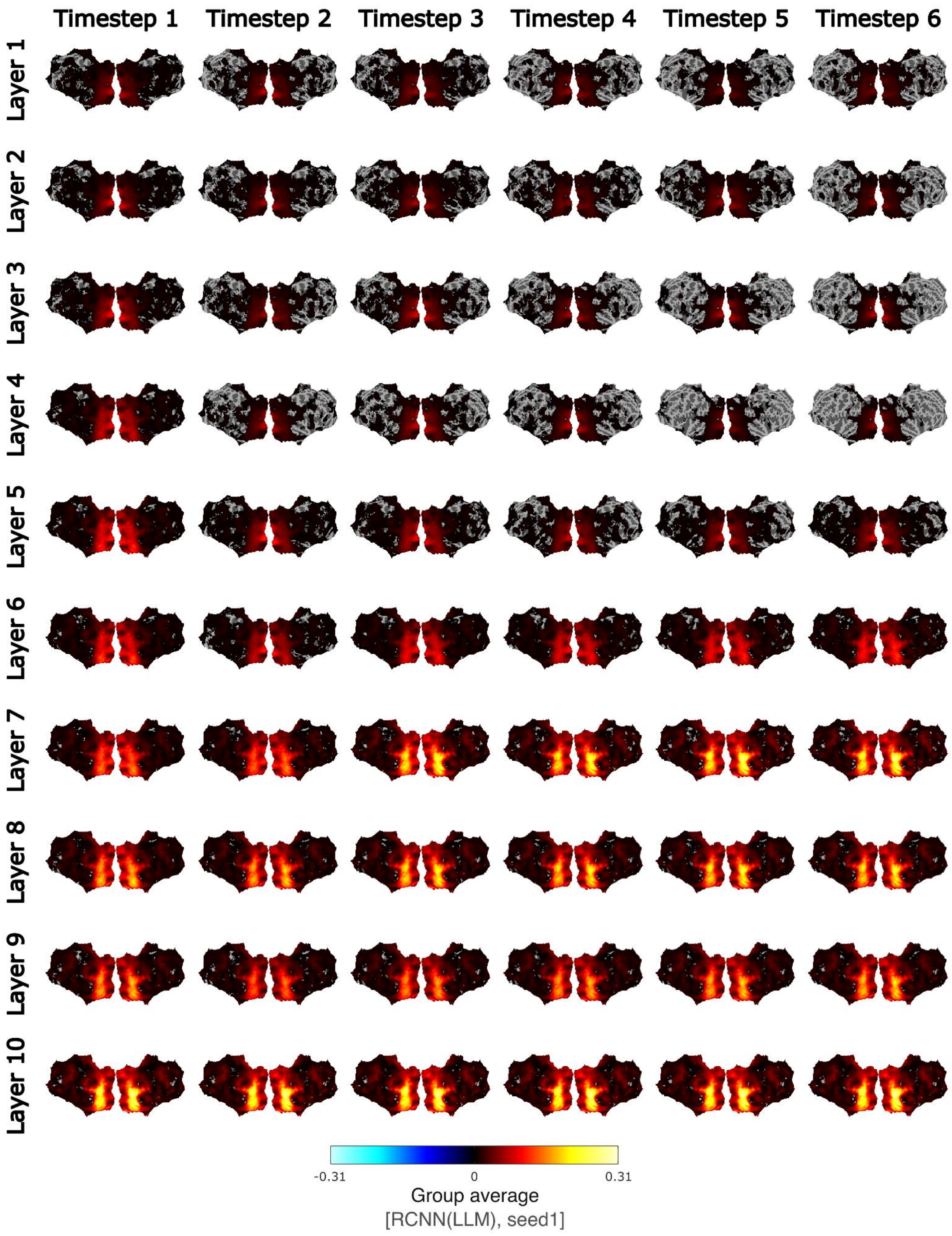

Supp. Figure 14: LLM-trained RCNN searchlight for all layers and timesteps. Pearson correlation (not noise-ceiling corrected) with brain data between the LLM-trained RCNN (using only the network trained with random seed 1, to show the performance of a single network; other plots in the paper always show correlations with RDMs averaged across 10 network instances). Statistics are computed as 2-tailed *t*-tests across subjects (N=8), separately for each layer/timestep, and all group level maps are thresholded at $p < 0.05$ with Benjamini & Hochberg false detection rate correction. All flatmaps share the same colormap, shown at the bottom. Earlier layers better predict earlier visual areas, and later layers better predict higher visual areas.

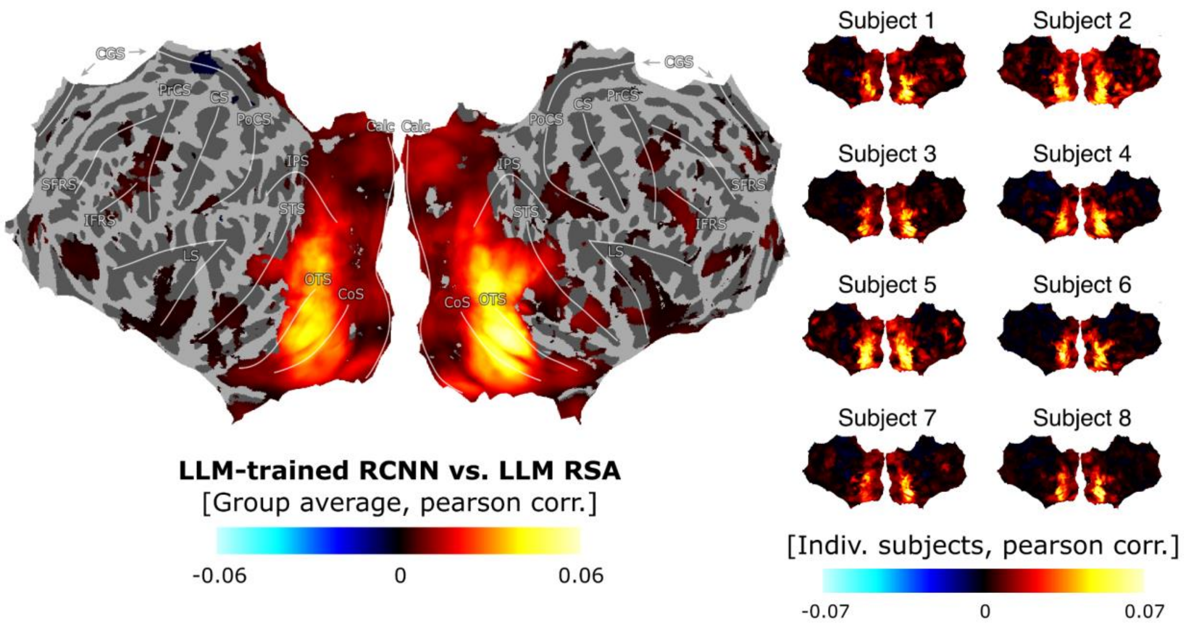

Supp. Figure 15: LLM-trained RCNN vs. LLM searchlight at the group- and subject-level. This is an extension of Fig. 4C. *Left*: Searchlight contrast for LLM-trained RCNN (last layer and timestep) vs. LLM embeddings. RCNN RDMs are averaged across 10 network instances; correlations are averaged across 8 participants for the group average; significance threshold set by a 2-tailed t-test across subjects (N=8) with Benjamini & Hochberg false discovery rate correction; p = 0.05. *Right*: individual subjects, not thresholded for significance.

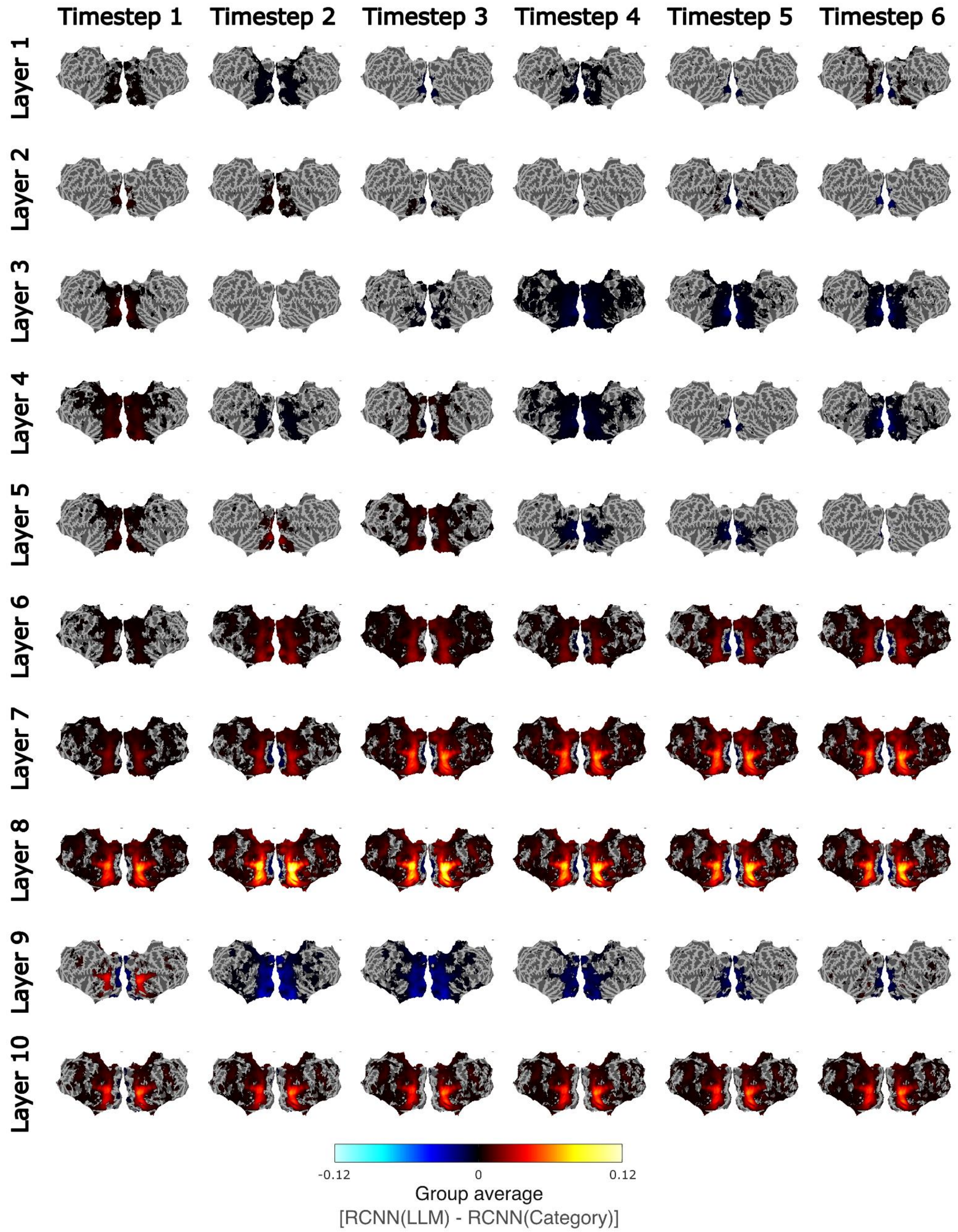

Supp. Figure 16: LLM-trained vs. category-trained RCNN searchlight contrasts for all layers and timesteps. Plots show the difference in Pearson correlation with brain data between the LLM-trained and category-trained RCNNs (red/yellow indicate an advantage for the LLM-trained RCNN, and blue indicates an advantage for the category-trained RCNN). Statistics are computed as 2-tailed *t*-tests across subjects (N=8), separately for each layer/timestep, and all group level maps are thresholded at $p < 0.05$ with Benjamini & Hochberg false detection rate correction. All flatmaps share the same colormap, shown at the bottom. In later layers (especially 6, 7, 8 &

10), the LLM-trained network has higher representational agreement with higher visual brain areas than the category-trained control. This effect is stronger after recurrence, especially in layer 7.

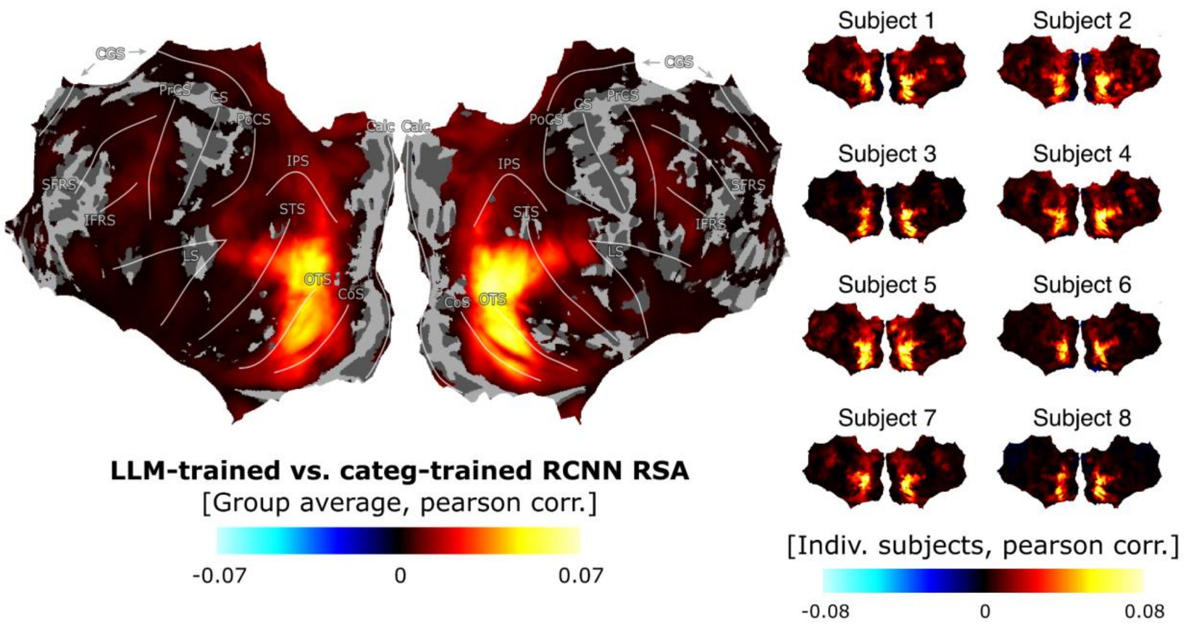

Supp. Figure 17: LLM-trained RCNN vs. category-trained RCNN searchlight at the group- and subject-level. This is an extension of Fig. 4D. *Left:* The flatmap shows the group level searchlight contrast (RCNN RDMs are averaged across 10 network instances; correlations are averaged across 8 participants; significance threshold set by a 2-tailed t-test across subjects with Benjamini & Hochberg false discovery rate correction; p = 0.05). *Right*: individual subjects, not thresholded for significance.

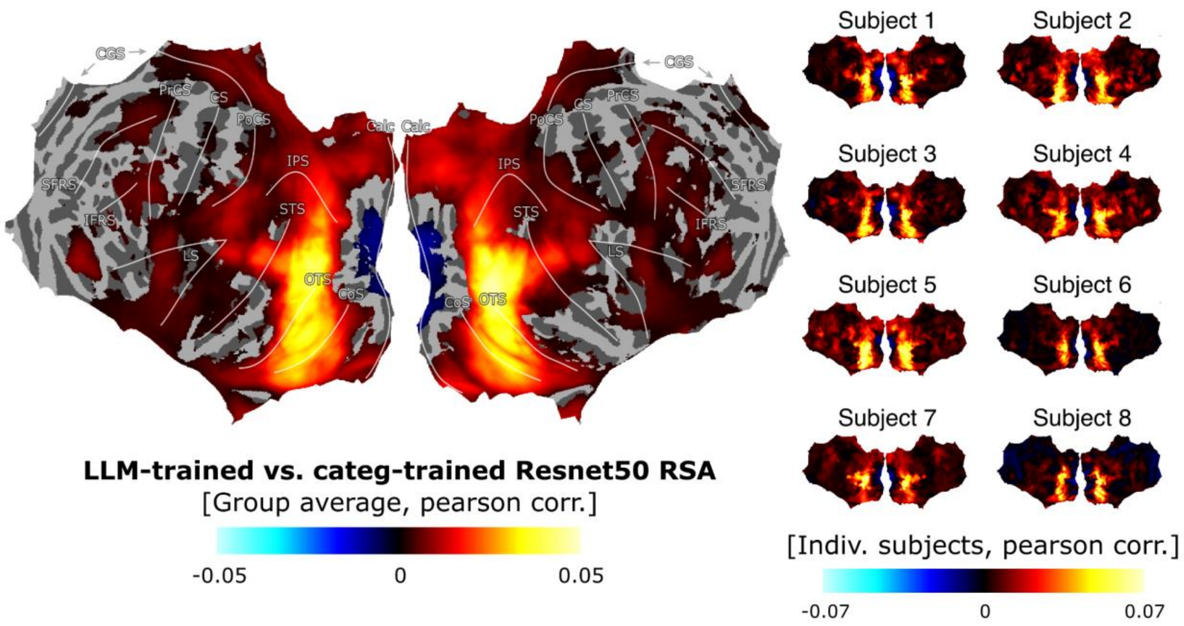

Supp. Figure 18: Reproducing the benefit in brain alignment of LLM training using ResNet50. We trained two identical ResNet50s from scratch in the same way as our RCNNs. One predicted LLM embeddings, and the other predicted multi-hot category vectors. *Left*: The flatmap shows the difference in Pearson correlation between the fMRI RDM and the pre-readout layer of these LLM- vs. category-trained ResNet50s at each searchlight location (red/yellow indicate an advantage for the LLM-trained ResNet50, and blue indicates an advantage for the category-trained ResNet50; correlations are averaged across 8 participants; significance threshold set by a 2-tailed t-test across subjects (N=8) with Benjamini & Hochberg false discovery rate correction; *p*=0.05). The LLM-trained ResNet50 outperforms the category-trained control in a widespread network of higher-level visual areas, reproducing our results obtained using RCNNs. *Right*: individual subjects, not thresholded for significance.

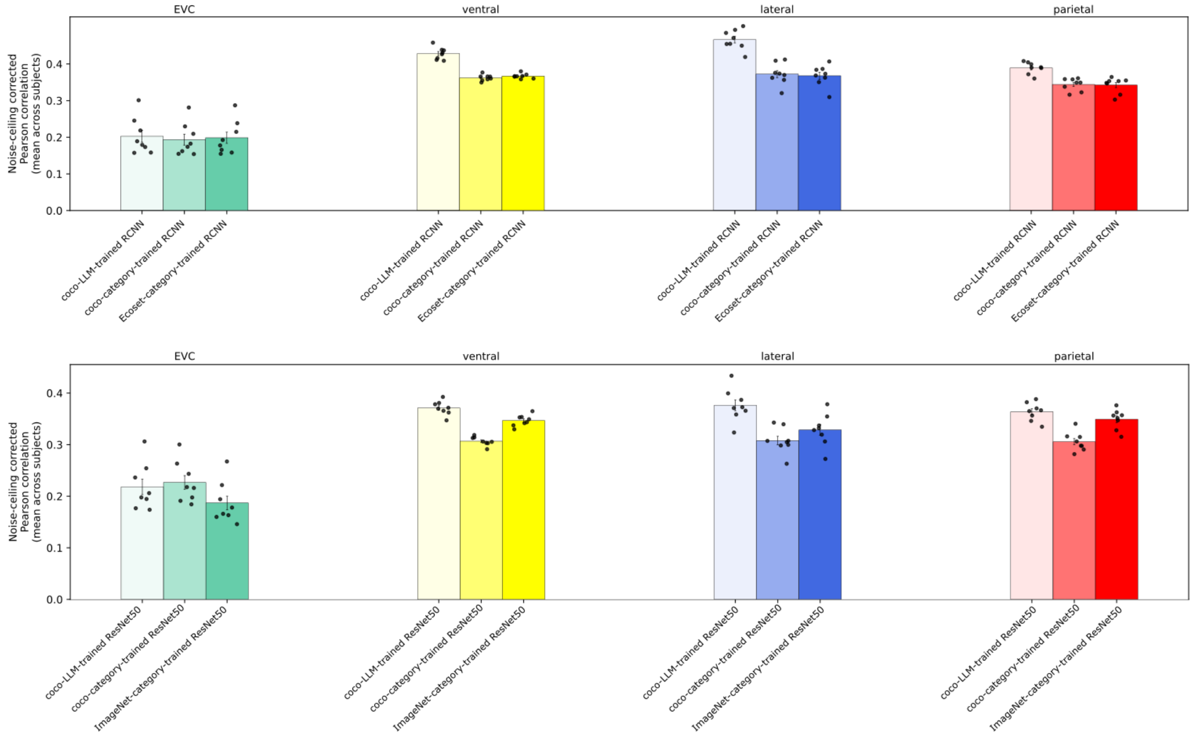

Supp. Figure 19: The advantage in brain alignment of our LLM-trained models is due to their training objective and not to the dataset. Our LLM-trained models are trained on the subset of COCO left after removing NSD images. To show that the good brain alignment of our models representations is due to LLM-training and not merely to the training on COCO, we applied the same ROI-wise RSA analysis as in Figs. 2 and 3E, and compared three models (top row): our main RCNN trained on COCO to predict LLM embeddings (coco-LLM-trained RCNN), the control RCNN trained on COCO to predict multi-hot category vectors (coco-category-trained RCNN), and an RCNN trained on ecoset to predict 1-hot category vectors (ecoset-category-trained RCNN; identical to "rcnn_ecoset" model of Fig. 4E). We also conducted the same comparison with ResNet50s instead of RCNNs (swapping ecoset for imagenet in for the last model, which is identical to the "resnet50" model of Fig. 4E; bottom row). In both cases, the networks trained to predict categories on COCO are similar or worse than networks trained to predict categories on ecoset or imagenet, showing that training on COCO is not enough to improve brain alignment compared to other training datasets. The coco-LLM-trained networks outperform all category-trained networks, showing that LLM-training, and not the COCO dataset, explains the good brain alignment of our models. Y-axis shows noise-ceiling corrected RDM correlations averaged across 8 participants; stars denote significant differences; significance threshold set by a 2-tailed t-test across subjects (N=8) with Benjamini & Hochberg false discovery rate correction; all significant *p*-values are <0.0005).

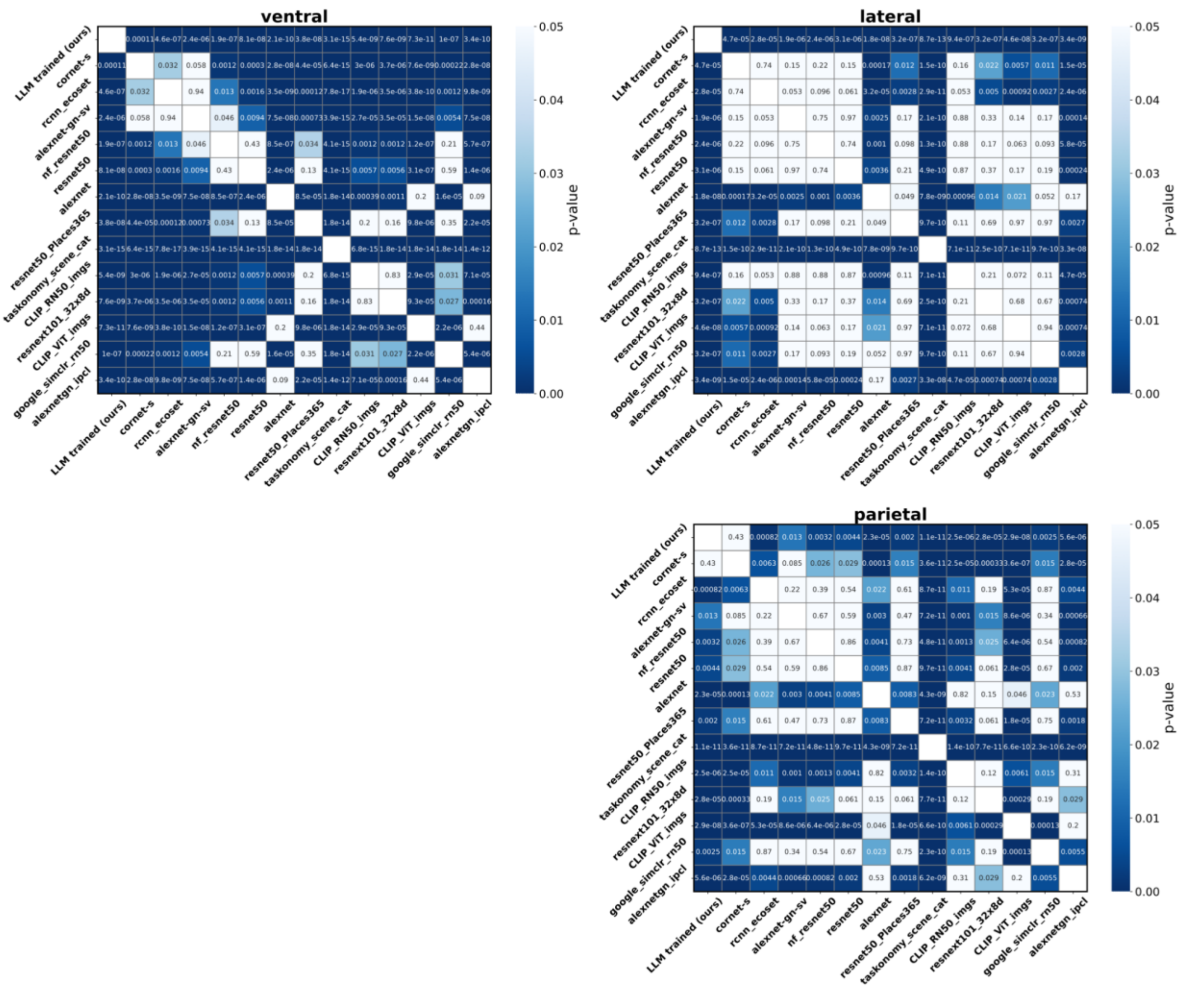

Supp. Figure 20: All Benjamin & Hochstein false discovery rate corrected p-values for Figure 4E. 2-tailed *t*-test across the 8 NSD participants. Corrections are conducted across all models, independently for each ROI.
